# A versatile *in vivo* platform for reversible control of transgene expression in adult tissues

**DOI:** 10.1016/j.stemcr.2024.11.003

**Published:** 2024-12-05

**Authors:** Jumpei Taguchi, Yosuke Yamada, Sho Ohta, Fumie Nakasuka, Takuya Yamamoto, Manabu Ozawa, Yasuhiro Yamada

**Affiliations:** 1 Core Laboratory for Developing Advanced Animal Models, Center for Experimental Medicine and Systems Biology, Institute of Medical Science, The University of Tokyo, Minato-ku, Tokyo 108-8639, Japan; 2 Department of Molecular Pathology, Graduate School of Medicine and Faculty of Medicine, The University of Tokyo, Bunkyo-ku, Tokyo 113-0033, Japan; 3 Department of Life Science Frontiers, Center for iPS Cell Research and Application (CiRA), Kyoto University, Kyoto 606-8507, Japan; 4 Institute for the Advanced Study of Human Biology (WPI-ASHBi), Kyoto University, Yoshida-Konoe-cho, Sakyo-ku, Kyoto 606-8501, Japan; 5 Medical-risk Avoidance Based on iPS Cells Team, RIKEN Center for Advanced Intelligence Project (AIP), Kyoto 606-8507, Japan

**Keywords:** Tet-ON system, Tet-OFF system, doxycycline, tetracycline, *In vivo* transgene expression, *In vivo* reprogramming, spacial and temporal regulation

## Abstract

Temporal control of transgenes has advanced biomedical interventions, including *in vivo* reprogramming, often utilizing the doxycycline (Dox)-mediated Tet-ON system. Here, we developed the Dox-mediated Tet-ON or complementary Tet-OFF counterpart to thoroughly investigate spatial and temporal transgene regulation in adult tissues, revealing inherent limitations and unexpected capabilities of each system. In stark contrast with the Tet-ON system, which was effective only in particular tissues and cell types, primarily epithelial cells, the Tet-OFF system proved capable of gene induction across diverse cell types. Despite the drawback of the Tet-OFF system in inducibility and tunability identified in our study, we demonstrated that use of tetracycline (Tc) effectively addresses these issues, possibly through its pharmacologic properties. Our data suggest that the Tc-mediated Tet-OFF system not only enables more versatile control of transgene expression but also offers a more biocompatible alternative for *in vivo* applications such as tissue regeneration and organismal rejuvenation.

## Highlights

• The *in vivo* Tet-ON system exhibits cell type-specific induction of transgene expression in adult mice
• The *in vivo* Tet-OFF system offers transgene induction in a wide variety of cell types in adult mice
• Tetracycline enables reversible and graded control of the transgene expression in the Tet-OFF system
• The tetracycline-mediated Tet-OFF system provides a versatile *in vivo* platform for controllable transgene expression

## Graphical abstract

**Figure:**
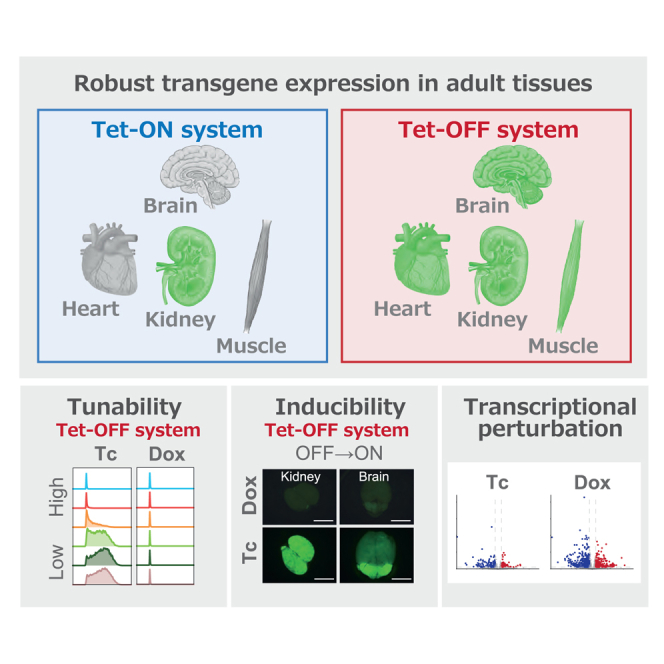

## Teaser

In this article, Yamada and colleagues investigate transgene regulation in adult mouse tissues with the doxycycline (Dox)-mediated Tet-ON or complementary Tet-OFF system. The Tet-OFF system enables gene induction in a broader spectrum of cell types while exhibiting limited inducibility and tunability. Finally, the authors show versatile control of transgene expression *in vivo* with the tetracycline (Tc)-mediated Tet-OFF system.

## Introduction

The Tet system was developed by Hermann Bujard and Manfed Gossen in 1992 based on the *Escherichia coli*-derived tetracycline (Tc) resistance operon to artificially control individual gene activities in mammalian cells (Gossen and Bujard, 1992; Wissmann et al., 1986). The genetic circuits consist of two elements: a Tc-responsive promoter (a minimal TATA-box containing a eukaryotic promoter fused with a Tet operator [tetO]) and a modified version of Tet repressor (TetR). The Tet-OFF system employs a Tc transactivator (tTA), a TetR protein fused with a VP16 transcription activation domain, whereas the Tet-ON system harnesses a reverse Tc transactivator (rtTA), which was discovered through random mutagenesis (Gossen et al., 1995). In the Tet-OFF system, tTA binds to tetO and thereby induces downstream transgene expression, while doxycycline (Dox), a Tc derivative, inhibits this binding, which halts transgene expression. By contrast, rtTA binds to tetO in the presence of Dox, leading to induction of transgene expression. Therefore, gene activity can be reversibly controlled in the Tet system by Dox exposure. Moreover, the level of transgene expression is tunable in the Tet-ON system by modulating the Dox concentration. Beyond transcriptional regulation, the Tet system enables perturbation of genome sequences, epigenetic modifications, and signal transductions when combined with CRISPR-Cas systems (Doudna and Charpentier, 2014; Jo et al., 2019), epigenetic regulators (Linhart et al., 2007), and constitutively active or dominant negative forms of signal transduction molecules, respectively.

Genetically engineered animal models have been employed to ectopically induce gene expression *in vivo* (Brinster et al., 1981; Costantini and Lacy, 1981; Gordon and Ruddle, 1981; Jaenisch and Mintz, 1974). Transgene expression can be induced in a cell type-specific manner when combined with genetic systems such as Cre/*loxP* recombination. Moreover, the timing of transgene induction is controllable with a tamoxifen-responsible recombinase (Brocard et al., 1997). However, these strategies constitutively induce transgene expression, which hampers their application to transiently perturb transcriptional regulation, as represented by the reprogramming process. Similarly, it is difficult to tune the levels of transgene expression. Although the Tet system has been widely used *in vitro*, previous studies developed *in vivo* animal models equipped with the Tet system (Beard et al., 2006; Gossen et al., 1995; Hochedlinger et al., 2005). Notably, the controllable nature of transgene expression with the Tet system has enabled the molecular basis of various physiological and pathological phenomena to be explored at the organismal level (Baron and Bujard, 2000; Berens and Hillen, 2003; Corbel and Rossi, 2002; Stieger et al., 2009). For instance, reversible expression of Yamanaka reprogramming factors in mice provided the proof of concept for an anti-aging strategy (Lu et al., 2020; Ocampo et al., 2016; Ohta and Yamada, 2023; Taguchi and Yamada, 2017) and uncovered the impact of epigenetic regulation on cancer development (Ohnishi et al., 2014; Shibata et al., 2018; Taguchi et al., 2021).

Although *in vivo* Tet systems have recently emerged as powerful tools in a range of research fields (Das et al., 2016) and an attractive modality for gene therapy (Das et al., 2016), there remain technical hurdles and unresolved issues. For instance, the Tet-ON system fails to robustly express transgenes in the adult brain (Beard et al., 2006). While the Tet-OFF system has been employed to induce expression of transgenes in the brain (Furth et al., 1994; Stieger et al., 2009), detailed information regarding expression patterns in other organs is unavailable. Moreover, previous studies suggested that the *in vivo* Tet-OFF system requires a substantial period for transgene reactivation after withdrawal of Dox (Kassai et al., 2014). Therefore, the dynamics of transgene expression in the *in vivo* Tet systems have not been fully elucidated (Fedorov et al., 2001; Furth et al., 1994; Kistner et al., 1996). In this study, we comprehensively analyzed transgene expression in the Tet-ON and Tet-OFF systems in adult mice under the same experimental settings. We propose a versatile platform and protocol to induce expression of transgenes in adult somatic tissues.

## Results

### Organ-specific transgene expression in adult Tet-ON mice

**Figure 1:**
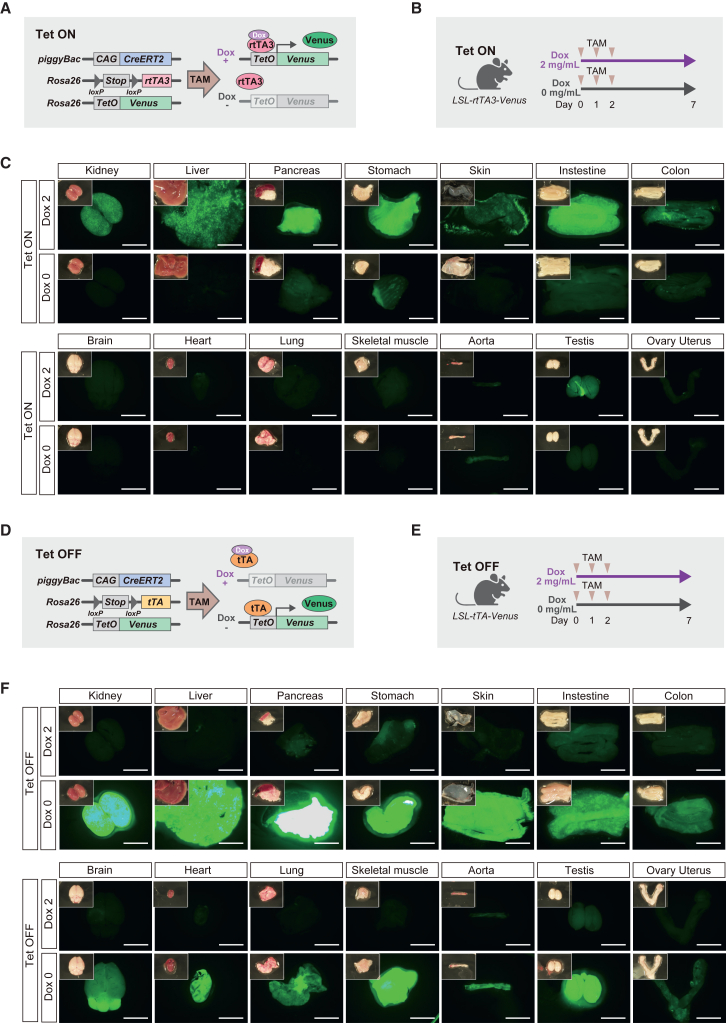
Macroscopic analysis of VENUS expression in Tet-ON/OFF mice (A) Schematic illustration of the *Venus* induction system in Tet-ON mice. (B) A protocol for *in vivo* induction of *Venus* expression in Tet-ON mice. (C) Representative macroscopic fluorescent images of organs in Tet-ON mice. Scale bars: 5 mm. (D) Schematic illustration of the *Venus* induction system in Tet-OFF mice. (E) A protocol for *in vivo* induction of *Venus* expression in Tet-OFF mice. (F) Representative macroscopic fluorescent images of organs in Tet-OFF mice. Scale bars: 5 mm.

To investigate transgene expression in an *in vivo* Tet-ON system, we generated a mouse model harboring a *piggyBac* (PB) *CAG*-*CreERT2* allele together with a *loxP-stop-loxP* (LSL)-*rtTA3* allele and a *tetO*-*Venus*-*ires*-*mCherry* allele at the *Rosa26* locus, a safe harbor locus that is permissive for transgene expression in all cell types (Figure 1A) (Soriano, 1999). In this model, tamoxifen treatment excises a stop cassette upstream of *rtTA3* (Das et al., 2004) through Cre/*loxP* recombination, which results in *rtTA3* expression, enabling the control of *Venus* expression with Dox (Figures 1A and 1B). We first obtained PB *CAG*-*CreERT2* mice with a *Rosa26*-*mTmG* reporter allele (Muzumdar et al., 2007) to assess the efficiency of Cre/*loxP* recombination after tamoxifen treatment (Figure S1A). EGFP signals were detected in all organs examined after tamoxifen administration (Figures S1B and S1C), indicating systemic recombination of *loxP* in adult PB *CAG*-*CreERT2* mice. Furthermore, tamoxifen administration induced *loxP* recombination and resultant *rtTA3* expression in a range of organs, including the brain, of Tet-ON mice (Figures S1D and S1E). When both tamoxifen and Dox (2 mg/mL in drinking water) were administered to Tet-ON mice at 4 weeks of age (Figure 1B), VENUS fluorescence was observed mainly in the kidneys, liver, pancreas, skin, and gastrointestinal tract (Figure 1C). Only autofluorescence was observed in the absence of Dox (Figure 1C). Some organs, such as the brain, heart, skeletal muscle, and lungs, did not exhibit detectable VENUS signals even in the presence of Dox (Figure 1C). Consistently, real time-qPCR revealed only modest expression of *Venus* mRNA in these organs (Figure S1F).

### Systemic induction of transgene expression in adult Tet-OFF mice

We next generated the Tet-OFF mouse model in which the *rtTA3* allele in the Tet-ON mouse was replaced by the *tTA* allele (Figure 1D). We confirmed efficient and systemic recombination of *loxP* after administration of tamoxifen in Tet-OFF mice (Figure S1G). Notably, in the absence of Dox, VENUS signals were observed in all organs examined, including the brain, heart, and lungs, in which the Tet-ON system failed to induce robust expression (Figures 1E and 1F). No detectable VENUS signal was observed after Dox treatment (2 mg/mL in drinking water) (Figure 1F), indicating that Dox tightly represses *Venus* expression.

### Cell type-specific transgene expression in adult Tet-ON mice

**Figure 2:**
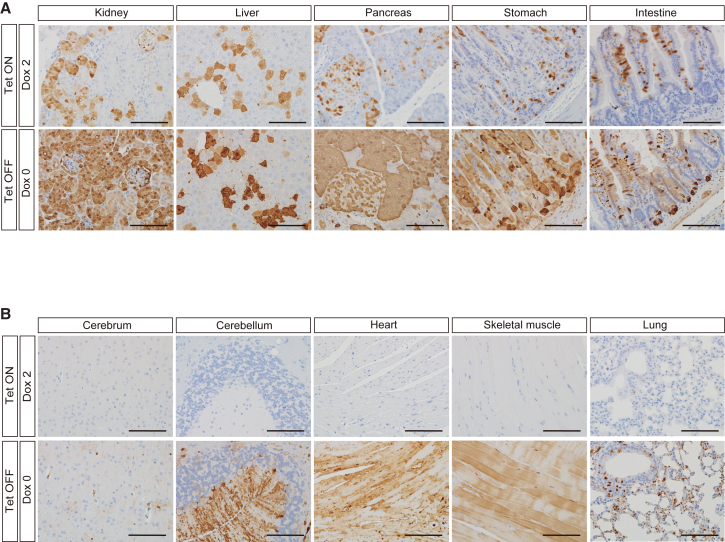
Microscopic analysis of VENUS expression in Tet-ON/OFF mice (A) Representative histological images of VENUS immunostaining. VENUS expression is observed in both Tet-ON and Tet-OFF mice. Scale bars: 100 μm. (B) Representative histological images of VENUS immunostaining. VENUS expression is detectable only in Tet-OFF mice. Scale bars: 100 μm.

**Figure 3:**
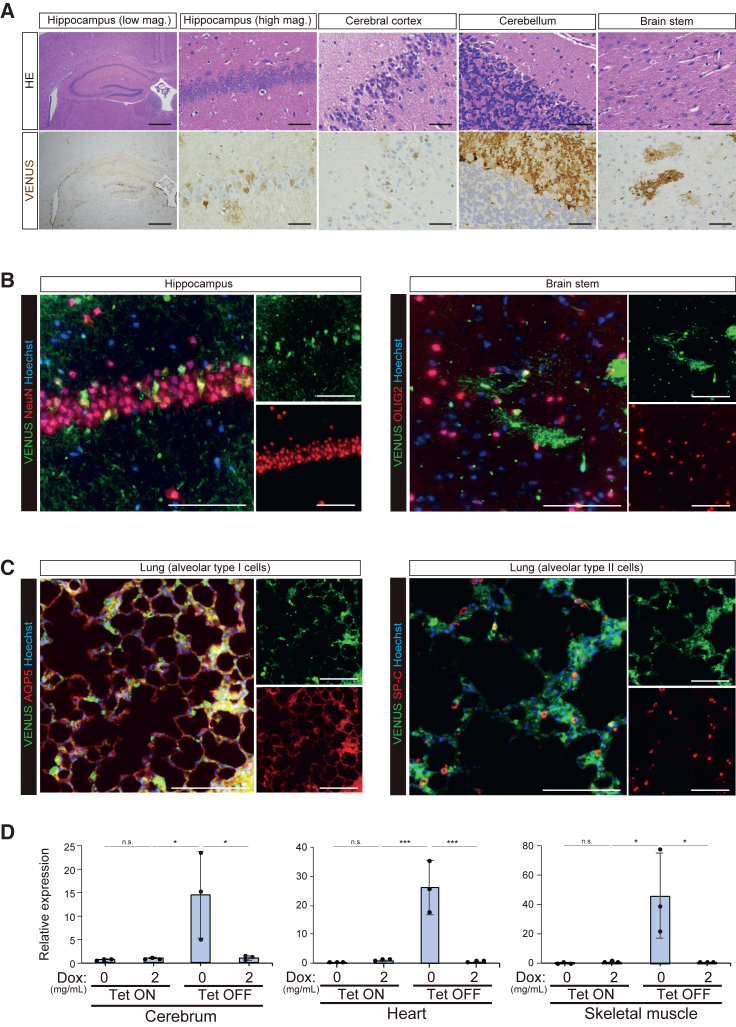
Broader expression of VENUS in Tet-OFF mice (A) Representative histological images of VENUS immunostaining in the central nervous system. Scale bars: 500 μm (hippocampus low), 50 μm (others). (B) Representative histological images of VENUS immunofluorescence staining in the hippocampus (left) and brain stem (right). NeuN-positive neuronal cells and OLIG2-positive glial cells express VENUS. Scale bars: 100 μm. (C) Representative histological images of VENUS immunofluorescence staining in the lungs. AQP5-positive alveolar type I cells and SP-C-positive alveolar type II cells express VENUS. Scale bars: 100 μm. (D) qPCR analysis of *Venus* expression in organs of Tet-ON and Tet-OFF mice. Data are presented as means ± SD of biological triplicates. Individual mice were used to perform biological triplicates. Expression levels relative to those in organs of Tet-ON mice administered tamoxifen and Dox are shown. Note that robust *Venus* expression is detected only in Tet-OFF mice. ^∗∗∗^*p* < 0.001, ^∗^*p* < 0.05; one-way ANOVA and Tukey’s multiple-comparison test.

Next, we performed immunohistological analysis to determine the cell types that express VENUS in Tet-ON mice (Figures 2, 3, S2, and S3; Table 1). An anti-GFP antibody that is also reactive with VENUS protein was utilized to detect VENUS expression (Taguchi et al., 2021). We first analyzed the organs in which macroscopic VENUS signals were detected (Figure 2A). In the kidneys of Tet-ON mice, VENUS-positive cells were mainly located in proximal tubules and glomeruli (Figures 2A and S3; Table 1). In the liver, hepatocytes and bile duct cells were positive for VENUS (Figures 2A and S3; Table 1). In the pancreas, acinar, duct, and islet cells exhibited VENUS expression (Figures 2A and S3; Table 1). In the stomach, VENUS expression was observed in the squamous epithelium in the forestomach and the glandular epithelium but was absent in the muscle layer (Figures 2A, S2A, and S3; Table 1). Similarly, in the small intestine and colon, VENUS was detected in epithelial cells in villi and crypts but was undetectable in the muscle layer (Figures 2A and S3; Table 1). In the skin, positive cells were observed in the epidermis and hair follicles (Figures S2A and S3; Table 1), but VENUS was not detected in mesenchymal cells in the dermis. In the testes, Leydig cells exhibited signals, whereas VENUS expression was not observed in mature germ cells (Figures S2B and S3; Table 1). Consistent with the lack of macroscopic signals, VENUS expression was not detected in any cell type in the brain, heart, smooth muscle, skeletal muscle, and lungs, except for the cerebral choroid plexus epithelium in the brain and a subset of bronchial epithelial cells in the lungs (Figures 2B and S3; Table 1). VENUS expression was not observed in Tet-ON mice without Dox administration (Figure S3). These results demonstrate that the Tet-ON system offers Dox-dependent transgene expression mostly in epithelial cells, but not in the majority of cell types in adult mice.

**Table 1:** VENUS expression in organs of Tet-ON/OFF adult mice

	Organ/tissue	Cell types	Tet-ON Dox(+)			Tet-OFF Dox(−)		
			Mouse 1	Mouse 2	Mouse 3	Mouse 1	Mouse 2	Mouse 3
Nervous system	cerebrum	neuron	–	–	–	+	++	+
		glial cells	–	–	–	+	++	+
	brainstem	neuron	–	–	–	++	++	+
		glial cells	–	–	–	+	++	+
	choroid plexus	epithelium	+	+	+	+++	+++	++
	cerebellum	molecular layers	–	–	–	+++	+++	++
		granular layers	–	–	–	+	++	+
		white matter	–	–	–	++	++	++
		Purkinje cells	–	–	–	+	++	+
Circulatory system	heart	cardiomyocytes	–	–	–	+++	+++	++
	vessel	endothelial cells	–	–	–	++	++	+
		smooth muscle	–	–	–	++	++	+
Respiratory system	lung	typeⅠ alveolar epithelium	–	–	–	+	++	+
		typeⅡ alveolar epithelium	–	–	–	+	++	+
		bronchial epithelium	+	+	+	++	++	++
Gastrointestinal system	stomach	cardia squamous epithelium	++	+	+	+	+	++
		pyloric glandular epithelium	++	+	NA	++	+	++
		muscle layers	–	–	–	+++	+++	++
		intermuscular plexus	–	–	–	+++	+++	++
	small intestine	epithelium	++	+	+	++	++	++
		muscle layers	–	–	–	++	+++	+
	colon	epithelium	+	+	+	++	++	++
		muscle layers	–	–	–	++	+++	++
	liver	hepatocytes	++	++	++	++	+++	++
		bile ducts	+	+	+	++	++	+
	pancreas	acinus	++	++	++	+++	+++	+++
		pancreatic islets	++	+	++	+++	++	++
		pancreatic ducts	+	+	+	++	+++	++
Urinary system	kidney	glomerulus	+	+	+	+	+++	++
		proximal tubules	++	++	++	+++	+++	++
		distal tubules	+	+	+	+++	+++	++
		collecting tubules	+	++	++	+++	+++	++
Epidermal system	skin	epidermis	+	+	+	+++	+++	++
		sebaceous glands	–	+	+	++	++	++
		hair follicles	+	++	+	+++	+++	++
		mesenchymal cells	–	–	–	++	++	++
Connective tissue	adipose tissues	white adipocytes	–	–	–	+	++	+
		brown adipocytes	+	–	+	++	++	++
	muscle	skeletal muscle	–	–	–	+++	+++	+
		smooth muscle	–	–	–	++	+++	+
Immune system	thymus	cortical epithelium	++	+	+	+	+	+
		medullary epithelium	++	+	+	+++	+++	+
		immature T cells	++	+	+	+	+	+
	spleen	megakaryocytes	++	+	+	++	++	+
		mononuclear cells in the white pulp	++	+	+	+	++	+
		mononuclear cells in the red pulp	++	+	+	+	++	+
			Mouse 1	Mouse 2	–	Mouse 1	Mouse 2	–
Reproductive system (male)	testis	spermatogonia	+	+	–	+	+	–
		sperm	–	–	–	–	–	–
		Leydig cells	+++	++	–	+++	+++	–
Reproductive system (female)	ovary	oocytes	–	–	–	–	–	–
		granulosa cells	–	–	–	+	+	–
		stromal cells	–	–	–	++	+	–
	uterus	intimal epithelium	+	+	–	+	+	–
		intimal stroma	–	–	–	+	+	–

Venus-positive cell rate. −: 0%, +: <10%, ++: 10%–50%, +++: >50%.

### Induction of transgene expression in diverse cell types in adult Tet-OFF mice

We then histologically analyzed VENUS expression in Tet-OFF mice (Figures 2, 3, S2, and S4; Table 1). Cell types that were permissive for induction of VENUS expression in Tet-ON mice also expressed VENUS in Tet-OFF mice in the absence of Dox (Figures 2A, S2A, and S4; Table 1). Semi-quantitative immunofluorescence analyses revealed that the expression levels in these cell types were comparable between the Tet-ON and Tet-OFF systems (Figures S5A and S5B). However, expression levels varied among cells and individuals in both systems (Figures S5A and S5B).

In sharp contrast with Tet-ON mice, VENUS was expressed in neuronal and glial cells of the cerebrum in Tet-OFF mice (Figures 2B and S4; Table 1). Neuronal cells in both the cerebral cortex and hippocampus displayed positive staining (Figures 3A and 3B). Additionally, in the cerebellum, VENUS was detected in the molecular and granular layers, white matter, and Purkinje cells (Figures 2B, 3A, and S4; Table 1). In the heart, VENUS expression was observed in cardiomyocytes and smooth muscle cells in coronary vessels (Figures 2B and S4; Table 1). VENUS expression was also detected in skeletal muscle and white adipose tissue (Figures 2B, S2B, and S4; Table 1). Notably, in the gastrointestinal tract, smooth muscle cells and peripheral nerve cells in the muscle layer as well as mesenchymal cells in the lamina propria exhibited VENUS expression (Figures S2A and S4; Table 1). Additionally, mesenchymal cells in the dermis were positive for VENUS (Figures S2A and S4; and Table 1). In the lungs, alveolar and bronchial epithelial cells were positively stained, in addition to a subset of mesenchymal cells (Figures 2B, 3C, and S4; Table 1). In the aorta, smooth muscle cells displayed positive staining (Figures S2B and S4; Table 1). In the spleen, a subset of immune cells in both red and white pulp displayed VENUS expression (Figures S2B and S4; Table 1). We did not observe detectable differences in the level or cell type specificity of the VENUS expression between males and females. In the ovaries, granulosa and stromal cells exhibited VENUS expression, although no obvious VENUS signal was detected in oocytes (Figures S2B and S4; Table 1). In the testes, spermatogonia and Leydig cells expressed VENUS. However, VENUS expression was undetectable in sperm (Figures S2B and S4; Table 1). VENUS expression was also confirmed at the mRNA levels in the cerebrum, heart, and skeletal muscle of Tet-OFF mice, while *Venus* mRNA was only slightly upregulated in Tet-ON mice, albeit without significant differences (Figure 3D). These results demonstrate that the Tet-OFF system offers transgene expression in most cell types in adult tissues.

### *In vivo* Tet system for transgene expression during early development

We next examined transgene expression during early development. Tet-ON and Tet-OFF zygotes were obtained by *in vitro* fertilization (Figures S5C and S5D). Upon development, VENUS fluorescence emerged as early as the morula stage in both Tet-ON and Tet-OFF embryos when they were cultured with and without Dox, respectively (Figure S5E). In blastocysts, inner cell mass and trophectoderm cells exhibited VENUS expression in both systems (Figure S5E). By contrast, VENUS fluorescence was not observed in Tet-ON and Tet-OFF embryos cultured without and with Dox, respectively (Figure S5E), indicating that both the Tet-ON and OFF systems work properly in preimplantation embryos.

We next transplanted Tet-ON and Tet-OFF zygotes into the uteri of pseudopregnant mice to allow their development (Figure S5D). In contrast with adult mice, Tet-ON embryonic day (E)14.5 embryos exhibited VENUS fluorescence throughout their bodies after host mice were administered Dox (Figure S5E), suggesting that embryonic cells are generally permissive for transgene induction even with the Tet-ON system. Tet-OFF E14.5 embryos similarly displayed systemic VENUS signals, which were abolished by administering Dox to host mice (Figure S5E). Immunostaining confirmed that VENUS was expressed in most cell types in both Tet-ON and OFF embryos treated with and without Dox, respectively (Figure S5F). Obvious VENUS fluorescence was observed in Tet-OFF placentae, while Tet-ON placentae exhibited only faint VENUS signals (Figures S5E and S5G), which provides additional evidence that the Tet-OFF system offers transgene expression in a broader spectrum of cell types. Consistently, VENUS expression in placental endothelial cells was exclusively detected in Tet-OFF mice (Figure S5G).

### Dox concentration-dependent graded control of transgene expression in the *in vivo* Tet-ON system

**Figure 4:**
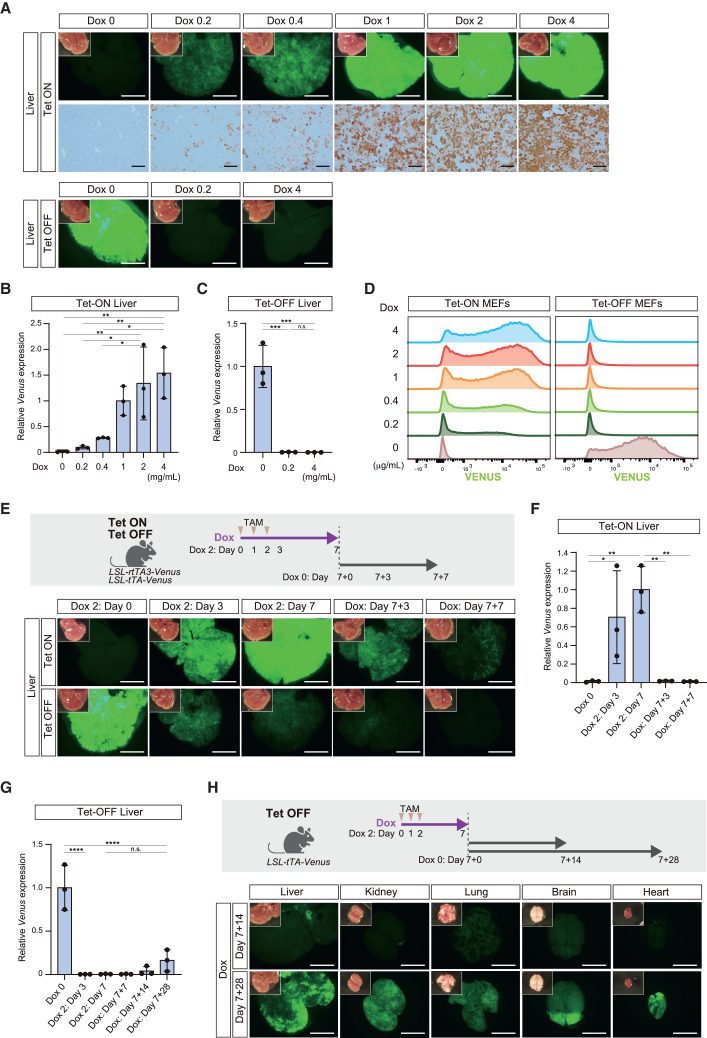
Dynamics of VENUS induction in Dox-treated Tet-ON/OFF mice (A) Representative macroscopic and microscopic images of VENUS expression in the liver of Dox-treated Tet-ON/OFF mice. Graded induction of VENUS expression depending on the Dox concentration is observed only in Tet-ON mice. Scale bars: 5 mm (macroscopic images), 200 μm (microscopic images). (B) qPCR analysis of *Venus* expression in the liver of Tet-ON mice. Data are presented as means ± SD of biological triplicates. Individual mice were used to perform biological triplicates. Expression levels relative to those in Tet-ON mice treated with 1 mg/mL Dox are shown. ^∗∗^*p* < 0.01, ^∗^*p* < 0.05; one-way ANOVA and Tukey’s multiple-comparison test. (C) qPCR analysis of *Venus* expression in the liver of Tet-OFF mice. Data are presented as means ± SD of biological triplicates. Individual mice were used to perform biological triplicates. Expression levels relative to those in Tet-OFF mice not administered Dox are shown. ^∗∗∗^*p* < 0.001, one-way ANOVA and Tukey’s multiple-comparison test. (D) Histogram of VENUS expression in Tet-ON/OFF MEFs exposed to different concentrations of Dox. Flow cytometric analysis reveals graded control of VENUS expression at the single-cell level in Tet-ON MEFs. (E) Reversible control of VENUS induction in the liver by Dox. An experimental protocol is shown in the upper panel. Note that reversible VENUS expression is observed only in Tet-ON mice. Scale bars: 5 mm. (F) qPCR analysis of *Venus* expression in the liver of Tet-ON mice. Data are presented as means ± SD of biological triplicates. Individual mice were used to perform biological triplicates. Expression levels relative to those in Tet-ON mice at day 7 are shown. ^∗∗^*p* < 0.01, ^∗^*p* < 0.05; one-way ANOVA and Tukey’s multiple-comparison test. (G) qPCR analysis of *Venus* expression in the liver of Tet-OFF mice. Data are presented as means ± SD of biological triplicates. Individual mice were used to perform biological triplicates. Expression levels relative to those in Tet-OFF mice not administered Dox are shown. ^∗∗∗∗^*p* < 0.0001; one-way ANOVA and Tukey’s multiple-comparison test. (H) Reversible expression of VENUS in the liver of Tet-OFF mice. An experimental protocol is shown in the upper panel. VENUS reactivation is detected weeks after Dox withdrawal. Scale bars: 5 mm.

Dosage control of transgene expression is an advantage of the Tet system *in vitro*. To investigate whether the levels of transgene expression are controllable in the *in vivo* Tet system, Tet-ON and Tet-OFF mice at 4 weeks of age were treated with different concentrations of Dox (0, 0.2, 0.4, 1, 2, and 4 mg/mL in drinking water for Tet-ON mice and 0, 0.2, and 4 mg/mL for Tet-OFF mice) and VENUS expression in the liver was analyzed at day 7. Tet-ON mice exhibited a gradual increase in VENUS fluorescence as the Dox concentration increased (Figure 4A). Consistently, VENUS immunohistochemistry revealed Dox concentration-dependent increases in both the staining intensity and positive cell area (Figure 4A). Stepwise increases in *Venus* mRNA expression were also confirmed (Figure 4B). In contrast with the graded regulation in the Tet-ON system, no detectable VENUS signal was observed in the Tet-OFF system even in mice treated with a lower concentration of Dox (0.2 mg/mL), which was also confirmed by the lack of *Venus* mRNA expression (Figures 4A and 4C).

To further quantitatively investigate the response to Dox, we established mouse embryonic fibroblasts (MEFs) with the Tet systems. Tet-ON MEFs exhibited Dox concentration-dependent increases in the level of VENUS fluorescence at the single-cell level in flow cytometric analysis (Figure 4D). However, VENUS signals remained suppressed in Tet-OFF MEFs treated with a lower concentration of Dox (0.2 μg/mL) (Figure 4D). These results demonstrate that the *in vivo* Tet-OFF system fails to control the levels of transgene expression within the range of concentrations adopted in previous experimental settings for the Tet systems (Tables S1 and S2).

### Reversible control of transgene expression in the *in vivo* Tet-ON system

We next tested the reversible control of transgene expression in the Tet system. Tamoxifen-treated Tet-ON and Tet-OFF mice at 4 weeks of age were treated with Dox (2 mg/mL in drinking water) for 7 days (day 7), which was subsequently withdrawn for 7 days (day 7 + 7), and then the dynamics of VENUS signals in the liver were examined (Figure 4E). In Tet-ON mice, VENUS fluorescence progressively increased during the first 7 days (Figure 4E). By contrast, VENUS signals progressively decreased upon Dox withdrawal and almost disappeared at day 7 + 7 (Figure 4E). The level of *Venus* mRNA increased and decreased more quickly than the level of VENUS fluorescence (Figure 4F), presumably reflecting the lower speed of translation and stability of VENUS protein, respectively. Thus, the *in vivo* Tet-ON system rapidly responds to Dox, which enables reversible control of transgene expression.

In Tet-OFF mice, VENUS signals rapidly decreased upon Dox treatment (2 mg/mL in drinking water) and were almost absent at day 7 (Figure 4E). However, upon withdrawal of Dox, reactivation of VENUS signals was undetectable at day 7 + 7 (Figure 4E). Consistently, *Venus* mRNA was almost undetectable at 3 days after Dox treatment (day 3) and remained undetectable at day 7 + 7 (Figure 4G), indicating that transgene expression was rapidly suppressed but not promptly reactivated in the Tet-OFF system. When we further extended the period of Dox withdrawal, VENUS fluorescence emerged in some organs at 2 weeks (day 7 + 14) and was increased at 4 weeks after withdrawal of Dox (day 7 + 28) (Figure 4H). However, VENUS signals and *Venus* mRNA expression were not fully recovered even at day 7 + 28 (Figures 4G and 4H). Collectively, these results demonstrate that transgene reactivation requires substantially longer in Tet-OFF mice.

### Tc enables graded and reversible gene activation in the *in vivo* Tet-OFF system

**Figure 5:**
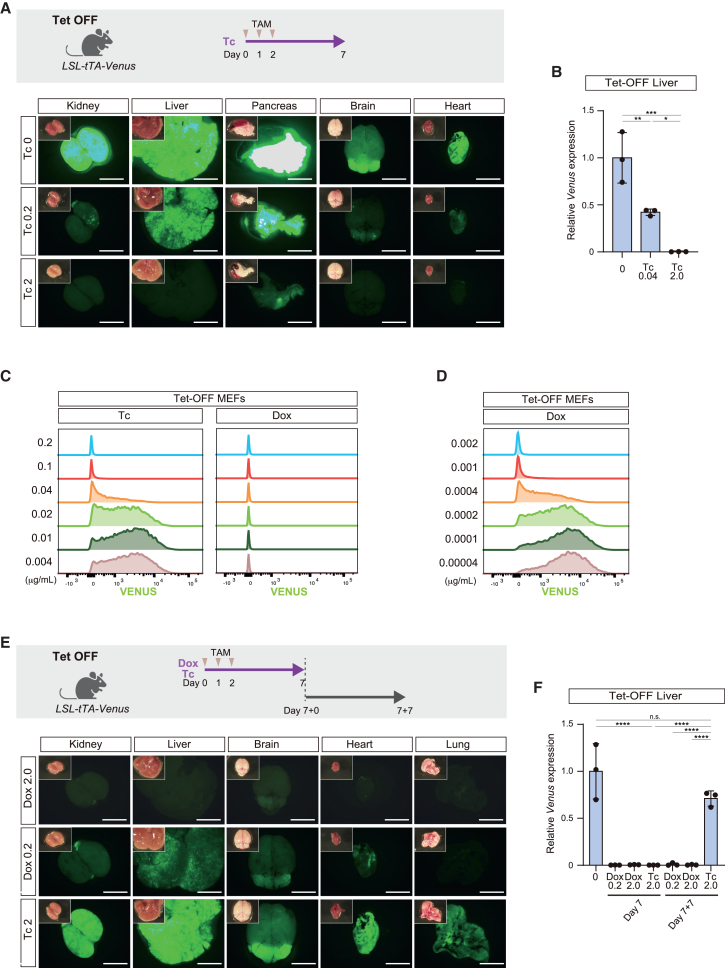
Rapid, reversible, and graded control of VENUS expression in Tc-treated Tet-OFF mice (A) Graded expression of VENUS in organs of Tc-treated Tet-OFF mice. An experimental protocol is shown in the upper panel. Scale bars: 5 mm. (B) qPCR analysis of *Venus* expression in the liver of Tet-OFF mice. Data are presented as means ± SD of biological triplicates. Individual mice were used to perform biological triplicates. Expression levels relative to those in Tet-OFF mice not administered Dox are shown. ^∗∗∗^*p* < 0.001, ^∗∗^*p* < 0.01, ^∗^*p* < 0.05; one-way ANOVA and Tukey’s multiple-comparison test. (C) Histogram of VENUS expression in Tet-OFF MEFs exposed to different concentrations of Tc and Dox. Flow cytometric analysis reveals graded control of VENUS expression in Tc-treated MEFs. (D) Histogram of VENUS expression in Tet-OFF MEFs exposed to a lower concentration of Dox. A substantially lower concentration of Dox is required for the activation of VENUS expression in Tet-OFF MEFs. (E) Reversible expression of VENUS in organs of Tet-OFF mice. An experimental protocol is shown in the upper panel. Rapid VENUS reactivation is detected after Tc withdrawal. Scale bars: 5 mm. (F) qPCR analysis of *Venus* expression in the liver of Tet-OFF mice. Data are presented as means ± SD of biological triplicates. Individual mice were used to perform biological triplicates. Expression levels relative to those in Tet-OFF mice not administered Dox are shown. ^∗∗∗∗^*p* < 0.0001; one-way ANOVA and Tukey’s multiple-comparison test.

Previous studies utilized Tc to control the Tet systems (Gossen and Bujard, 1992; Gossen et al., 1995; Krueger et al., 2004). Tc has a much lower affinity for TetR than Dox (Degenkolb et al., 1991). Considering this weak affinity of Tc, we tried to achieve graded control of transgene expression by administering lower concentrations of Tc (Figure 5A). To this end, we treated Tet-OFF mice with 0.2 mg/mL Tc in drinking water. In contrast with the tight repression observed upon treatment with 0.2 mg/mL Dox, Tc-treated mice exhibited sustained but reduced VENUS signals at day 7 (Figures 5A and 5B), suggesting that transgene expression was partially repressed *in vivo*. To further investigate the graded control, Tet-OFF MEFs were treated with different concentrations of Tc. The VENUS intensity was inversely correlated with the concentration of Tc (Figure 5C), demonstrating that Tc achieves graded control of transgene expression. By sharp contrast, Dox exposure strictly repressed VENUS signals even at a concentration of 4 ng/mL (Figure 5C). VENUS signals started to appear at a concentration of 400 pg/mL Dox, indicating that the graded control of transgene expression could be achieved even with Dox at substantially lower concentrations in the Tet-OFF system (Figure 5D). Together, our results demonstrate that Tc enabled graded transgene activation within the range of concentrations adopted in previous studies with the Tet systems.

Finally, to overcome the limitation in reversible control of transgene expression in the *in vivo* Tet-OFF system, we first tested a reduced concentration of Dox (0.2 mg/mL in drinking water) (Figure 5E). Administration of 0.2 mg/mL Dox was sufficient to repress VENUS fluorescence and *Venus* mRNA expression in the liver of Tet-OFF mice at day 7. However, only modest reactivation of VENUS fluorescence and *Venus* mRNA expression was observed after the Dox withdrawal (day 7 + 7) (Figures 5E and 5F). Importantly, Tc has a significantly shorter half-life in serum than Dox (Klein and Cunha, 1995). Therefore, with the aim of rapidly reactivating transgenes, we administered Tc instead of Dox to Tet-OFF mice (Figure 5E). We confirmed that administration of 2 mg/mL Tc in drinking water for 7 days was sufficient to repress VENUS signals and *Venus* expression in adult tissues of Tet-OFF mice (Figure 5A). We then examined transgene reactivation after withdrawal of Tc for 7 days. Tc-treated Tet-OFF mice exhibited reactivation of VENUS signals in most organs at a similar level as control mice (Figure 5E), supporting the assumption that Tc is rapidly cleared *in vivo*. Robust and rapid reactivation of VENUS expression was also confirmed at the mRNA level (Figure 5F). In summary, we leveraged the pharmacologic advantages of Tc to achieve reversible and graded control of transgene expression in Tet-OFF mice.

### Transcriptional perturbation caused by Dox and Tc

**Figure 6:**
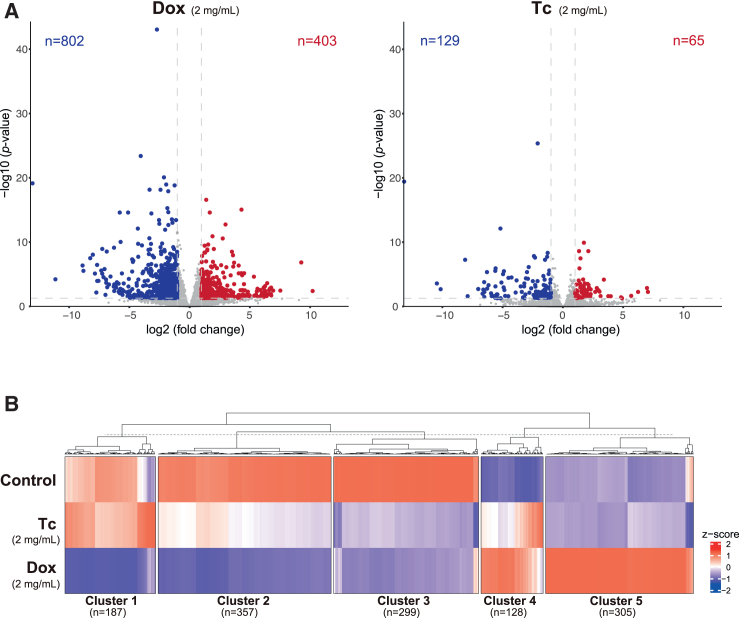
Transcriptional perturbation caused by Dox and Tc administration (A) Volcano plots of RNA-seq data showing the transcriptional response to Dox or Tc administration. The data represent the mean values of 3 independent samples. Differentially expressed genes (fold change > 2, FDR < 0.05) are labeled in red or blue. (B) Heatmap of RNA-seq data. The data represent the mean values of 3 independent samples. Upregulated and downregulated genes (fold change > 2) after Dox or Tc administration were subjected to K-means clustering.

Dox and Tc may have unintended effects independent of tetO-regulated exogenous genes. Previous studies pointed out the potential disadvantage of using Dox by providing evidence that it impairs mitochondrial function (Moullan et al., 2015). To compare transcriptional perturbation by these agonists *in vivo*, we conducted RNA sequencing (RNA-seq) analyses of the liver of mice administered Dox or Tc in drinking water at a concentration of 2 mg/mL for 7 days. Consistent with previous studies, the expression levels of a subset of genes were altered after Dox treatment (Figures 6A and S6A). Remarkably, although Tc treatment also changed gene expression, the number of affected genes was much smaller than that in Dox-treated mice (Figure 6A). When changes were present in both Dox- and Tc-treated mice, the extent of these changes was generally smaller in the latter mice (Figure 6B). Notably, gene ontology analysis revealed that genes associated with the term “cellular response to external stimulus” were highly represented in the subset of genes specifically upregulated in Dox-treated mice (cluster 5) (Figure S6B). These results suggest that use of Tc or a low dose of Dox in the Tet-OFF system as an agonist mitigates transcriptional perturbation and potentially alleviates any harmful effects of Dox in the Tet-ON system, thereby improving the interpretation of experimental phenotypes.

## Discussion

The transcription network is stably maintained in somatic cells, which safeguards the homeostasis of tissues (Bulger and Groudine, 2011; Hnisz et al., 2013; Long et al., 2016). Accordingly, impairment of transcriptional regulation often results in cellular dysfunction, leading to impaired tissue functions that cause disease development. Therefore, genetic disruption and/or ectopic gene activation have been employed in animals to study gene functions in physiology and their significance in pathology. However, *in vivo* genetic ablation and transgene expression are generally irreversible, which limits the use of these strategies to study biological phenomena, especially those related to epigenetic regulation. For instance, embryonic development is a dynamic process, during which changes in the extracellular environment affect transcriptional regulation, leading to the stepwise organization of epigenetic modifications in order to ensure the spatiotemporal control of cell fate alterations. Indeed, environmental perturbations within a limited time window in embryos often cause latent pathologies in adults, as proposed by the DOHaD hypothesis (Barker, 2007). As such, temporal transcriptional alteration in response to the extracellular environment acts as an interface between environmental and epigenetic regulation that persists upon cell replication. Therefore, transient control of gene expression has advantages over static intervention to recapitulate and/or perturb such transcriptional dynamics. Here, we successfully achieved reversible and graded control of transgene expression in most cell types in adult tissues using the Tc-mediated *in vivo* Tet-OFF system. Of note, this inducible system can be applied to repress gene expression if combined with other technologies such as the CRISPR-dCas9-KRAB system (Thakore et al., 2015). Considering the critical role of the kinetic parameters of transcriptional regulation in diverse biological and pathological processes, the *in vivo* Tet-OFF platform should help to uncover the molecular basis of tissue homeostasis and diseases.

Successful reprogramming into induced pluripotent stem cells requires the silencing of transgenic Yamanaka factors (Takahashi and Yamanaka, 2006), indicating that temporal, not continuous, expression of reprogramming factors is crucial for this process. Somatic cells can be reprogrammed *in vivo* with the Tet-ON system (Abad et al., 2013; Ohnishi et al., 2014). Notably, partial reprogramming evoked by cyclic induction of reprogramming factors is effective for extending the lifespan of progeroid mice and regenerating tissues (Chen et al., 2021; Lu et al., 2020; Ocampo et al., 2016). Similarly, emerging evidence indicates that direct reprogramming, which bypasses the pluripotent state, provokes tissue regeneration (Hirano et al., 2022; Srivastava and DeWitt, 2016). Thus, transient induction of reprogramming factors *in vivo* has drawn significant attention as a promising strategy for tissue regeneration and rejuvenation. Here, we propose a versatile platform to induce gene activation in adult somatic tissues. Given that the Tet-ON system is not permissive for robust transgene expression in neuronal cells, cardiomyocytes, and skeletal muscle cells, all of which exhibit a limited regenerative potential, the devised system may offer a suitable platform for such interventions. Moreover, although *in vivo* reprograming-mediated lifespan extension has garnered considerable interest, the underlying mechanisms remain unclear. The cell type-specific nature of transgene expression in the *in vivo* Tet-ON system may offer important insights into the mechanisms underlying reprogramming factor-induced longevity. Additionally, the spatiotemporal atlas of transgene expression in this study provides valuable information for gene therapies with vectors equipped with the Tet systems (Chtarto et al., 2003).

In this study, we demonstrated that the *in vivo* Tet-ON system exhibits Dox-dependent, but cell type-specific, induction of transgene expression in adult mice, which is in line with observations in a previous study where the Tet system was knocked into a *Col1a1* locus for EGFP induction (Beard et al., 2006). These results suggest that the cell type specificity of the Tet-ON system is likely independent of target loci and gene cargoes. We did not observe a correlation between DNA methylation levels at the CMV promoter downstream of the Tet operator and expression levels of transgenes in the organs exhibiting variable VENUS expression levels in the Tet-ON system (Figure S6D). Although mechanisms underlying the cell type-specific expression remain unclear, other factors, such as differences in tetO binding ability in conjunction with the distinct affinity of Dox with tTA/rtTA in each cell type, might be responsible for the cell type-dependent reactivity.

Consistent with the fact that Tcs bind bacterial ribosomes, which are evolutionarily associated with mitochondrial ribosomes, Dox disturbs mitochondrial proteostasis in mammalian cells. Here, we show that Tc has limited effects on transcriptional profiles. Notably, mitochondrial component gene expression was altered more frequently in Dox-treated mice than in Tc-treated mice (Figure S6C), implying that Tc mitigates the mitochondrial detriments associated with use of Dox. Although the toxicity of Tc must be carefully considered when interpreting experimental outcomes, especially when it is administered for a long period (Moullan et al., 2015), our data suggest that the Tc-mediated Tet-OFF system offers a more biocompatible alternative than the Dox-mediated Tet-ON system.

Despite the robust gene activation in a broader range of cell types in the *in vivo* Tet-OFF system, there remain limitations. First, we failed to induce transgene expression in mature germ cells, which may be associated with unique epigenetic regulation in these cells (Sasaki and Matsui, 2008). Second, the level of transgene expression varied among cells and individuals even with identical genotypes, suggesting the presence of unidentified factors that affect transgene expression, which are presumably involved in epigenetic regulation. Finally, silencing of a transgene after longer induction as well as leaky expression, both of which have been observed in the *in vivo* Tet-ON system (Zhu et al., 2011), should be carefully considered in practical application of the *in vivo* Tet-OFF system. Elucidation of the molecular mechanisms underlying the variable induction of transgene expression will pave the way toward further improvement of the transgenic system *in vivo*, which will contribute not only to diverse biomedical research but also to efficient gene therapies for diseases as well as age-related detrimental phenotypes.

## Experimental procedures

### Establishment of ESCs

#### PB CAG-CreERT2 ESCs

A PB transposon vector carrying *CAG-CreERT2-ires-NeoR* and a PB transposase plasmid (pCAG-PBase [Kim et al., 2016]) were electroporated into V6.5 ESCs (C57BL/6 × 129/sv) using the Neon transfection system (Thermo Fisher Scientific). After selection with 350 μg/mL G418 (Nacalai Tesque), G418-resistant ESC colonies were picked and expanded to establish ESC lines.

#### *Rosa26-LSL-rtTA3/tTA* ESCs

*Rosa26-LSL-rtTA3*/*tTA* ESCs were generated according to a previously described protocol (Ozawa et al., 2022). Briefly, a circular *Rosa26-LSL-rtTA3*/*tTA* vector and *Rosa26* Cas9-ribonucleoprotein (RNP), which is composed of gRNA targeting a *Rosa26* locus (IDT) and Alt-R S.p. Cas9 Nuclease V3 (IDT), were electroporated into V6.5 ESCs using the Neon transfection system. The sequence of the gRNA targeting the *Rosa26* locus is as follows: CGCCCATCTTCTAGAAAGAC. After selection with 350 μg/mL G418, G418-resistant ESC colonies were picked and expanded to establish ESC lines.

#### *Rosa26-tetO-Venus-ires-mCherry* ESCs

*Rosa26-tetO-Venus-ires-mCherry* ESCs were generated according to a previously described protocol (Ozawa et al., 2022). Briefly, a circular *Rosa26-tetO-Venus-ires-mCherry* vector and *Rosa26* Cas9-RNP, which is composed of gRNA targeting a *Rosa26* locus and Alt-R S.p. Cas9 Nuclease V3, were electroporated into V6.5 ESCs using the Neon transfection system. After selection with 15 μg/mL blasticidin S (Funakoshi), blasticidin-resistant ESC colonies were picked and expanded to establish ESC lines.

### Flow cytometric analysis

Cells were washed with phosphate-buffered saline (PBS) and incubated in 0.25% trypsin-EDTA (Nacalai Tesque) for 5 min at 37°C. After centrifugation at 200*g* for 3 min, cell pellets were resuspended in fluorescence-activated cell sorting (FACS) buffer (PBS containing 4% bovine serum albumin [BSA]) and passed through a cell strainer. VENUS-positive cells were analyzed using an FACSCanto II instrument (BD). Flow cytometric data were analyzed using FlowJo V10 (BD).

### Mice

All animal experiments were approved by the Animal Experiment Committee at IMSUT, and animal care was conducted in accordance with institutional guidelines. All mice were housed in a specific pathogen-free animal facility under a 12-h light/12-h dark cycle with food and water available *ad libitum*.

### Tamoxifen administration

Tamoxifen (Sigma) was dissolved in corn oil (Invitrogen) to a concentration of 20 mg/mL. Mice were intraperitoneally treated with 2 mg of tamoxifen once daily for three consecutive days.

### Dox and Tc administration

Four-week-old mice received Dox hyclate (Sigma) in drinking water supplemented with 10 mg/mL sucrose (Nacalai Tesque). Dox was added to CARD-KSOM medium used to maintain preimplantation embryos at a concentration of 2 μg/mL. To observe transgene expression at E14.5, pseudopregnant mice implanted with Tet-ON or Tet-OFF embryos were administered 2 mg/mL Dox. Tc hydrochloride (Wako) was administered to mice in the same manner as Dox.

### Histological analysis, immunostaining, and immunofluorescence

Dissected tissue samples were fixed in 4% paraformaldehyde (Nacalai Tesque) overnight at room temperature. Fixed samples were embedded in paraffin using HistoCore PEARL (Leica Biosystems). Sections were sliced at a thickness of 3–4 μm. Samples were soaked three times for 5 min each in lemosol (Wako) to remove paraffin and three times for 5 min each in 100% ethanol to hydrophilize. After washing with water for several minutes, samples were soaked in epitope-retrieval buffer (Nichirei Biosciences) and microwaved at 100 W for 10 min. Samples were then soaked in PBS for several minutes and incubated with 200 μL of primary antibodies diluted in PBS containing 2% BSA (MP Biomedicals) at 4°C overnight. A rabbit monoclonal anti-GFP primary antibody (Abcam, #ab183734, dilution 1/200) was used to detect VENUS protein. Sections were incubated with horseradish peroxidase-conjugated secondary antibodies (Nichirei Bioscience, Histofine) at room temperature for 30 min, and chromogen development was performed using DAB (Nichirei Bioscience). Stained slides were counterstained with hematoxylin. The primary antibodies used for immunofluorescence were chicken polyclonal anti-GFP (Abcam, #ab13970, dilution 1/1,000) to detect VENUS protein, rabbit monoclonal anti-NeuN (Abcam, #ab177487, dilution 1/1,000), rabbit monoclonal anti-OLIG2 (Abcam, #ab109186, dilution 1/100), mouse monoclonal anti-AQP5 (Santa Cruz, #sc-514022, dilution 1/100), rabbit monoclonal anti-SP-C (Abcam, #ab211326, dilution 1/500), and rabbit monoclonal anti-CK8 (Abcam, #ab53280, dilution 1/500). Sections were stained for 90 min at room temperature with the following secondary antibodies conjugated with fluorescent proteins diluted in PBS containing 2% BSA: CF488A anti-chicken immunoglobulin G (IgG) (Biotium, #20166, dilution 1/500), Alexa Fluor 555 anti-rabbit IgG (Invitrogen, #A-21429, dilution 1/500), and Alexa Fluor 555 anti-mouse IgG (Invitrogen, #A-31570, dilution 1/500). After two washes for 5 min in PBS, sections were mounted using ProLong glass antifade mountant with NucBlue stain (Invitrogen) and evaluated with a BZ-X710 fluorescence microscope (KEYENCE).

### Genomic DNA extraction and PCR analysis

Freshly collected tissues were incubated in tail lysis buffer (Nacalai Tesque) at 65°C for 2 h or longer. Genomic DNA was purified by phenol-chloroform extraction and ethanol precipitation and dissolved in TE buffer (Nacalai Tesque). Genomic DNA was quantified on a NanoDrop 2000 instrument (Thermo Fisher Scientific) and diluted to a concentration of 50 ng/μL. One microgram of genomic DNA was used for PCR analysis with KOD-FX-Neo.

### Statistics and reproducibility

All statistical parameters, including the statistical comparison test and exact *p* value, are described in the figures or figure legends. Statistical analyses were performed using the Prism 10 software (GraphPad). Data are presented as the means ± standard deviation (SD). The reproducibility of representative images was confirmed in a minimum of three independent biological samples.

## Resource availability

### Lead contact

Further information and requests for resources and reagents should be directed to and will be fulfilled by the lead contact, Yasuhiro Yamada (yyamada@m.u-tokyo.ac.jp).

### Materials availability

Materials generated in this study will be made available on request with a completed Materials Transfer Agreement. The animal strains generated in this study will be deposited at the RIKEN BioResource Research Center and Center for Animal Resources.

### Data and code availability

The Gene Expression Omnibus accession number for the RNA-seq data reported in this paper is GSE268589. All relevant data supporting the key findings of this study are available within the article and its supplemental information files or from the corresponding author upon reasonable request.

## Acknowledgments

We are grateful to M. Kikuchi, K. Miyazaki, N. Tako, R. Kimoto, M. Baba, T. Mashimo, and M. Ikawa for technical assistance. This study was supported by T. Ando in the Pathology Core laboratory and Y. Ishii and K. Ono in the FACS Core laboratory, The Institute of Medical Science, The 10.13039/501100004721University of Tokyo (IMSUT). Yasuhiro Yamada was supported in part by 10.13039/100009619AMED (23zf0127008h0002, 23tm0524004h0001, 233fa627001h0002, 23bm1223002h0002, 23bm1123040s0201, and 23ama221201h0002) and the 10.13039/501100001691JSPS KAKENHI (23H05485 and 23H00407). J.T. was supported by the 10.13039/501100001691JSPS KAKENHI (23K14114) and 10.13039/501100005865Mochida Memorial Foundation for Medical and Pharmaceutical Research. The Institute for the Advanced Study of Human Biology (ASHBi) is supported by the World Premier International Research Center Initiative (WPI), MEXT, Japan.

## Author contributions

J.T. and Yasuhiro Yamada designed and conceived the study. J.T., S.O., and Yasuhiro Yamada wrote the paper. J.T. performed the experiments. J.T., S.O., T.Y., and F.N. performed RNA-seq analysis. Yosuke Yamada supported histological analyses. M.O. provided technical instructions.

## Declaration of interests

The authors declare no competing interests.

## Supplemental information

Document S1. Figures S1–S6, Tables S1–S3, and supplemental experimental procedures

Document S2. Article plus supplemental information
