# Supplementary material for: A versatile in vivo platform for reversible control of transgene expression in adult tissues
Source: Stem Cell Reports. 2024 Dec 5;20(1):102373. doi: 10.1016/j.stemcr.2024.11.003 (PMC11784451; doi:10.1016/j.stemcr.2024.11.003)
Supplement: Document S1. Figures S1–S6, Tables S1–S3, and supplemental experimental procedures [file mmc1.pdf]

Stem Cell Reports, Volume *20*

Supplemental Information

A versatile *in vivo* platform for reversible control of transgene expression in adult tissues

Jumpei Taguchi, Yosuke Yamada, Sho Ohta, Fumie Nakasuka, Takuya Yamamoto, Manabu Ozawa, and Yasuhiro Yamada

A versatile *in vivo* platform for reversible control of transgene expression in adult tissues

Taguchi et al.

Inventory of Supplementary Information

Supplemental Figures and Legends (Figure S1-S6)

Supplemental Tables (Table S1-S3)

Supplemental Experimental Procedures

References

Figure S1

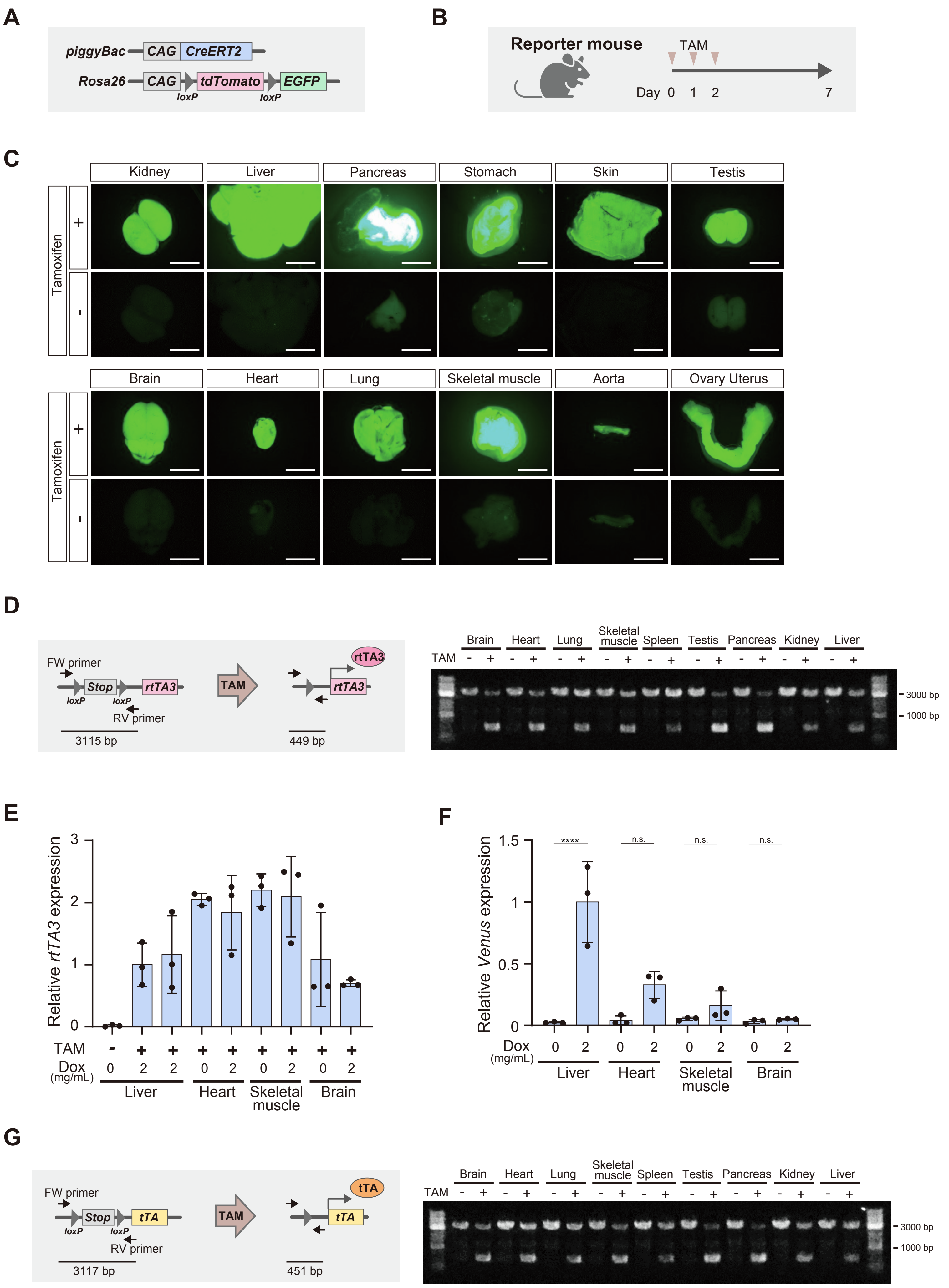
A
piggyBac
CAG
CreERT2
Rosa26
CAG
tdTomato
EGFP
loxP
loxP
B
Reporter mouse
TAM
Day 0 1 2 7
C
Kidney
Liver
Pancreas
Stomach
Skin
Testis
Tamoxifen
+
-
Brain
Heart
Lung
Skeletal muscle
Aorta
Ovary Uterus
D
FW primer
Stop
rtTA3
loxP
loxP
RV primer
3115 bp
TAM
rtTA3
449 bp
Brain
Heart
Lung
Skeletal muscle
Spleen
Testis
Pancreas
Kidney
Liver
TAM
3000 bp
1000 bp
E
Relative rtTA3 expression
TAM
Dox (mg/mL)
Liver
Heart
Skeletal muscle
Brain
F
Relative Venus expression
****
n.s.
Dox (mg/mL)
Liver
Heart
Skeletal muscle
Brain
G
FW primer
Stop
tTA
loxP
loxP
RV primer
3117 bp
TAM
tTA
451 bp
Brain
Heart
Lung
Skeletal muscle
Spleen
Testis
Pancreas
Kidney
Liver
TAM
3000 bp
1000 bp

Figure S2

A

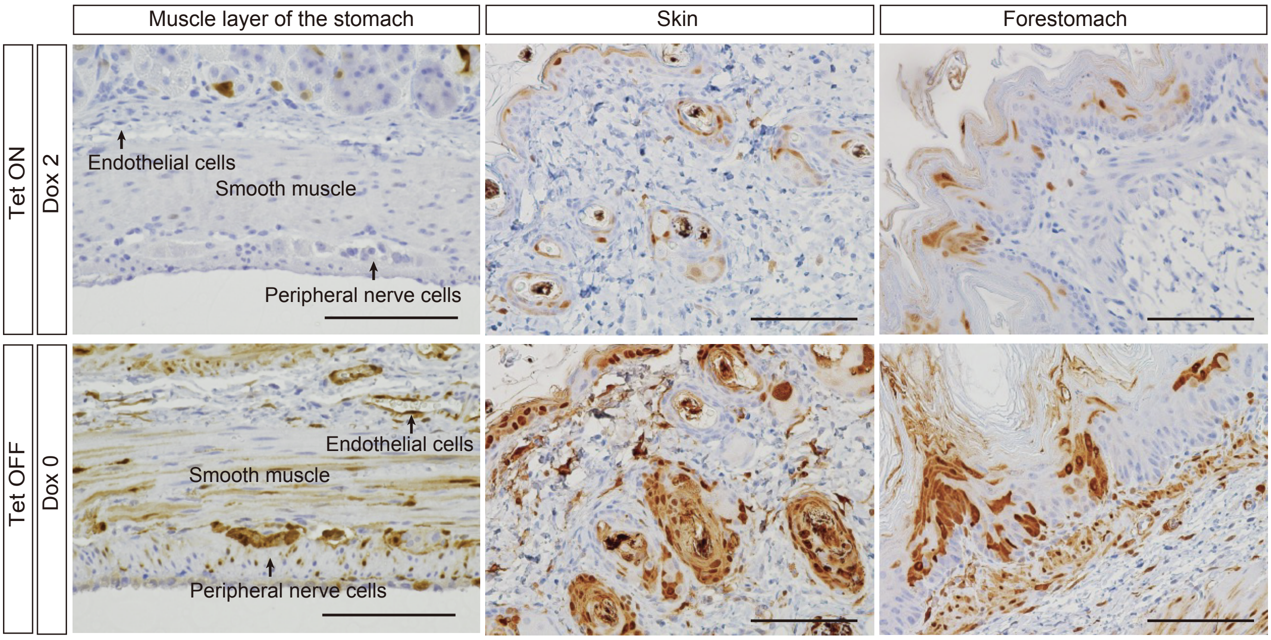

Muscle layer of the stomach
Skin
Forestomach
Tet ON
Dox 2
Tet OFF
Dox 0
Endothelial cells
Smooth muscle
Peripheral nerve cells
Endothelial cells
Smooth muscle
Peripheral nerve cells

B

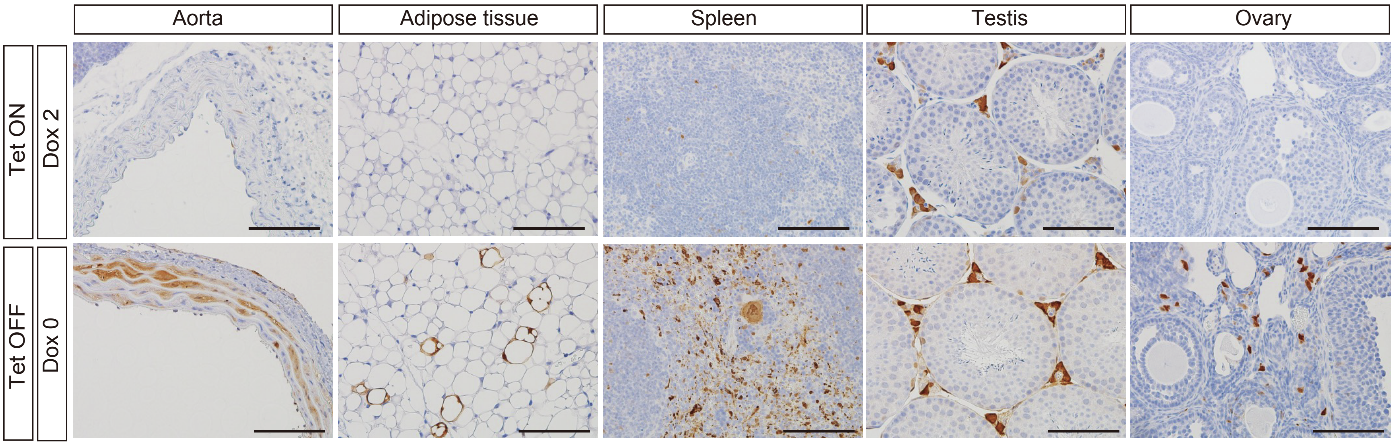

Aorta
Adipose tissue
Spleen
Testis
Ovary
Tet ON
Dox 2
Tet OFF
Dox 0

Figure S3

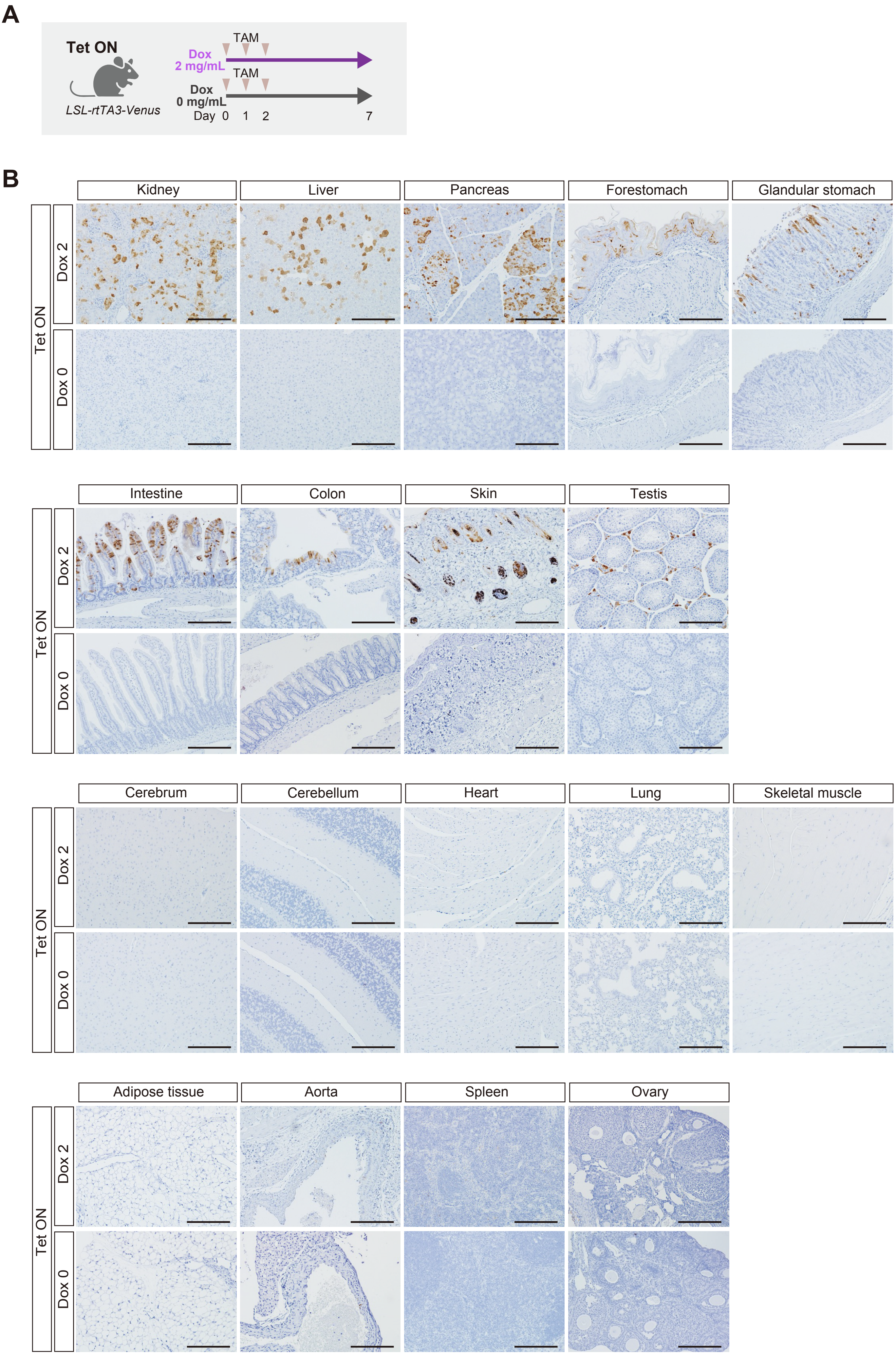
A
Tet ON
LSL-rtTA3-Venus
Dox
2 mg/mL
TAM
Dox
0 mg/mL
TAM
Day 0 1 2 7
B
Kidney
Liver
Pancreas
Forestomach
Glandular stomach
Intestine
Colon
Skin
Testis
Cerebrum
Cerebellum
Heart
Lung
Skeletal muscle
Adipose tissue
Aorta
Spleen
Ovary
Tet ON
Dox 2
Dox 0

Figure S4

A

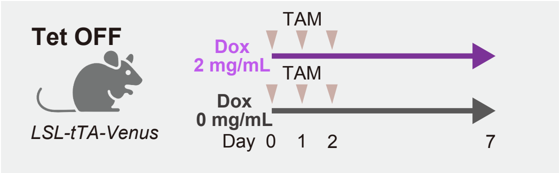

Tet OFF
LSL-tTA-Venus
TAM
Dox
2 mg/mL
TAM
Dox
0 mg/mL
Day 0 1 2 7

B

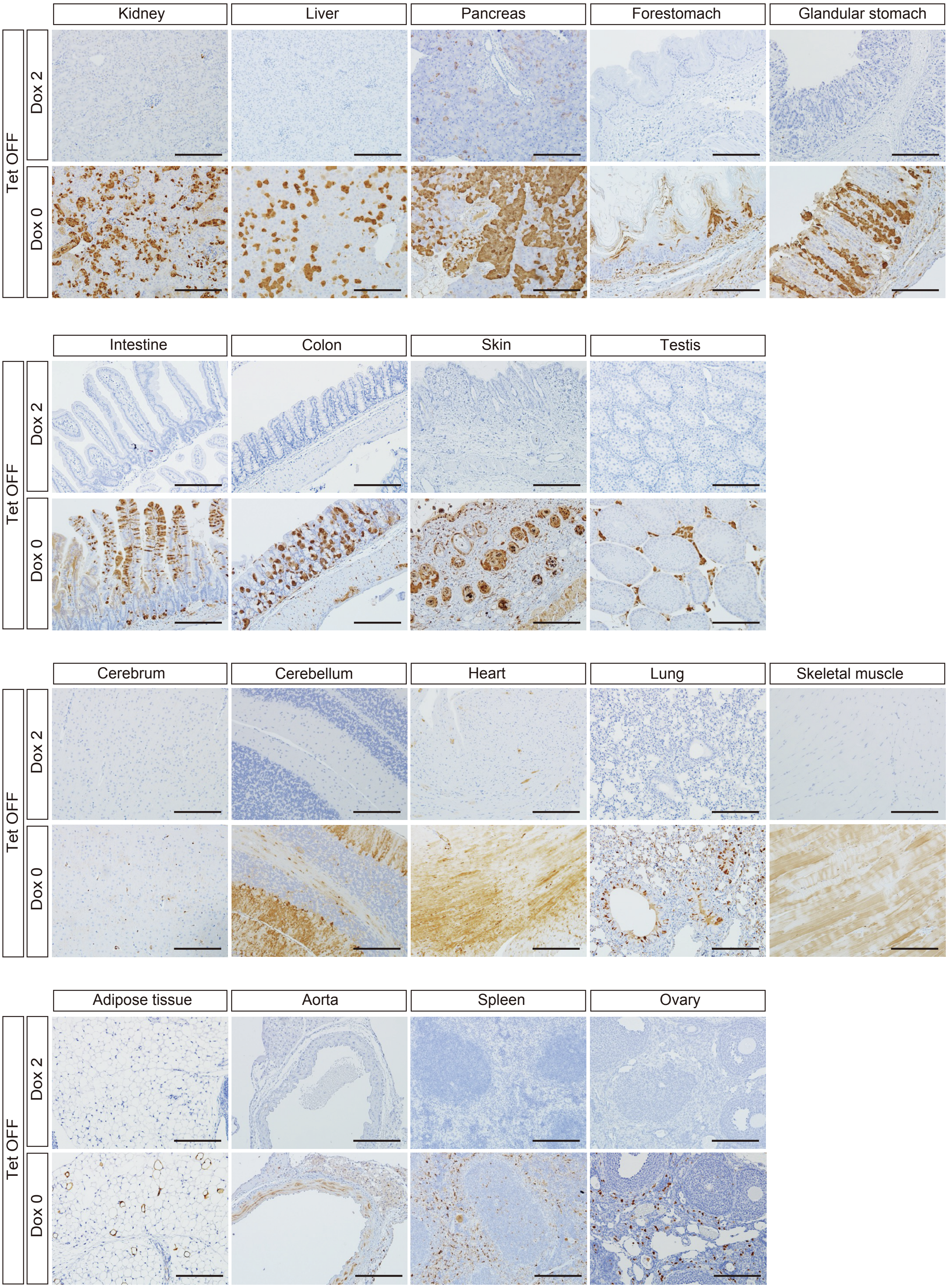

Kidney
Liver
Pancreas
Forestomach
Glandular stomach
Tet OFF
Dox 2
Dox 0
Intestine
Colon
Skin
Testis
Tet OFF
Dox 2
Dox 0
Cerebrum
Cerebellum
Heart
Lung
Skeletal muscle
Tet OFF
Dox 2
Dox 0
Adipose tissue
Aorta
Spleen
Ovary
Tet OFF
Dox 2
Dox 0

Figure S5

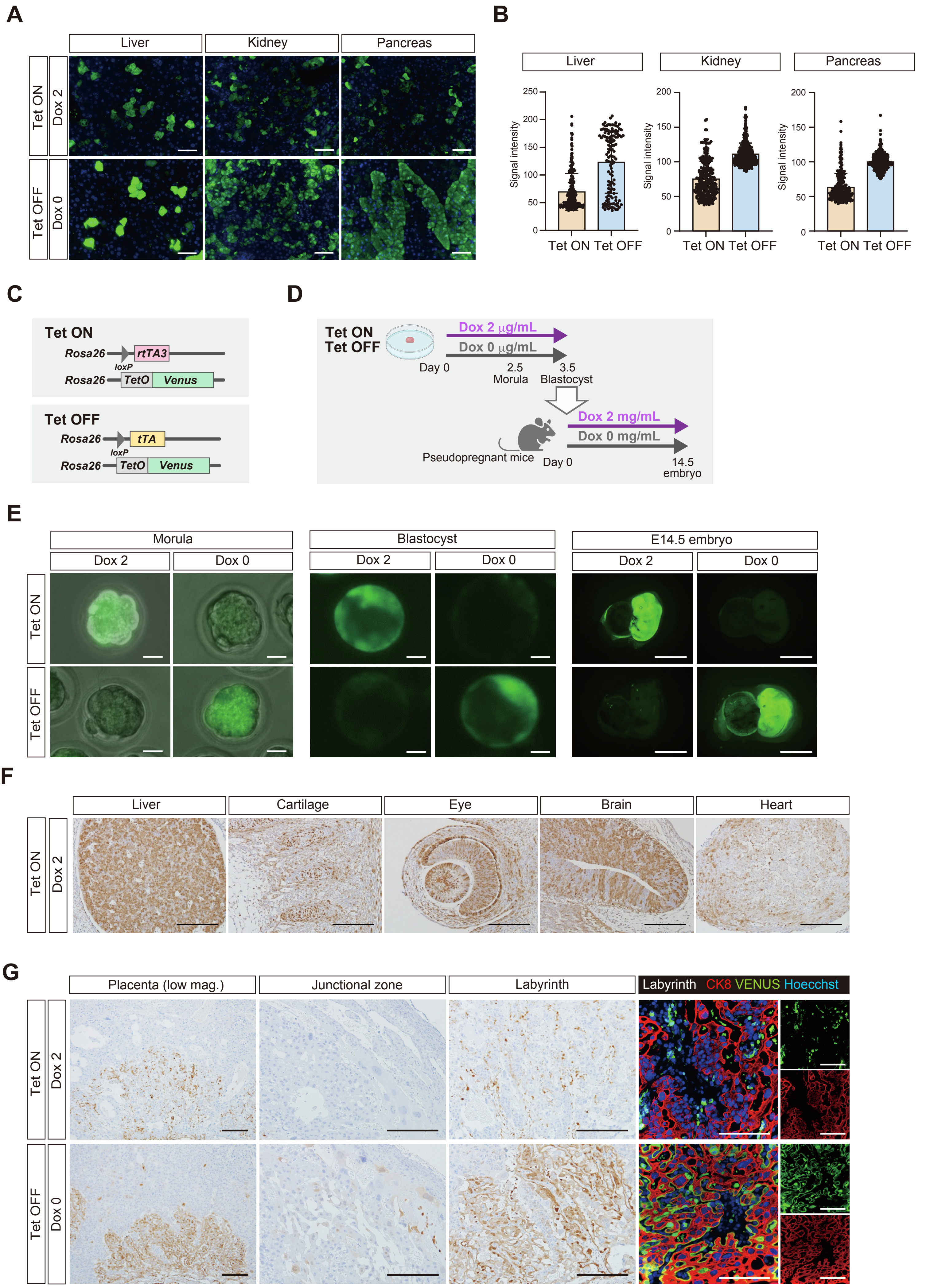
A
Liver
Kidney
Pancreas
Tet ON
Dox 2
Tet OFF
Dox 0
B
Liver
Kidney
Pancreas
Signal intensity
Tet ON Tet OFF
C
Tet ON
Rosa26
rtTA3
loxP
TetO
Venus
Tet OFF
tTA
D
Tet ON
Tet OFF
Dox 2 μg/mL
Dox 0 μg/mL
Day 0
2.5
Morula
3.5
Blastocyst
Dox 2 mg/mL
Dox 0 mg/mL
Pseudopregnant mice
14.5
embryo
E
Morula
Blastocyst
E14.5 embryo
Dox 2
Dox 0
F
Liver
Cartilage
Eye
Brain
Heart
G
Placenta (low mag.)
Junctional zone
Labyrinth
Labyrinth CK8 VENUS Hoecchst

Figure S6

A

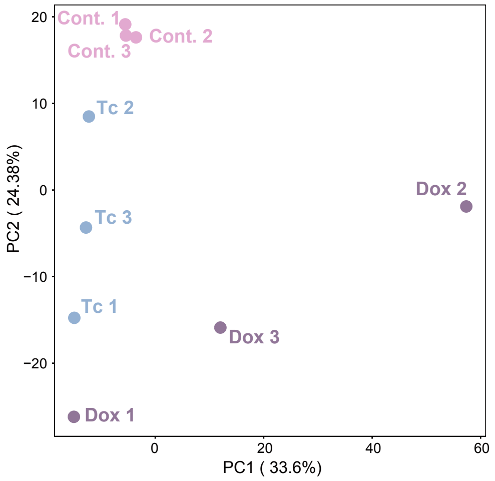

B

Cluster	GO accession	GO	p-value
1	GO:0006953	acute-phase response	2.67E-11
	GO:0002526	acute inflammatory response	1.90E-07
	GO:0007051	spindle organization	2.60E-07
	GO:0051988	regulation of attachment of spindle microtubules to kinetochore	3.04E-07
	GO:1902850	microtubule cytoskeleton organization involved in mitosis	3.86E-07
	GO:0007052	mitotic spindle organization	5.78E-07
2	GO:0006909	phagocytosis	6.03E-13
	GO:0032943	mononuclear cell proliferation	7.05E-13
	GO:0070661	leukocyte proliferation	1.08E-12
	GO:0007159	leukocyte cell-cell adhesion	4.59E-12
	GO:0042098	T cell proliferation	8.44E-11
	GO:0046651	lymphocyte proliferation	9.32E-11
3	GO:0007159	leukocyte cell-cell adhesion	2.56E-13
	GO:0046651	lymphocyte proliferation	1.03E-11
	GO:0032943	mononuclear cell proliferation	1.57E-11
	GO:0050900	leukocyte migration	1.71E-11
	GO:0070661	leukocyte proliferation	1.71E-11
	GO:0070663	regulation of leukocyte proliferation	5.76E-11
4	GO:0006631	fatty acid metabolic process	2.02E-08
	GO:0006641	triglyceride metabolic process	1.51E-07
	GO:0006639	acylglycerol metabolic process	1.25E-06
	GO:0006638	neutral lipid metabolic process	1.39E-06
	GO:0042178	xenobiotic catabolic process	1.41E-06
	GO:1904478	regulation of intestinal absorption	2.97E-06
5	GO:0071496	cellular response to external stimulus	1.04E-10
	GO:0031668	cellular response to extracellular stimulus	4.94E-10
	GO:0031669	cellular response to nutrient levels	2.64E-09
	GO:0009267	cellular response to starvation	6.86E-09
	GO:0042594	response to starvation	3.06E-08
	GO:0009991	response to extracellular stimulus	3.22E-08

C

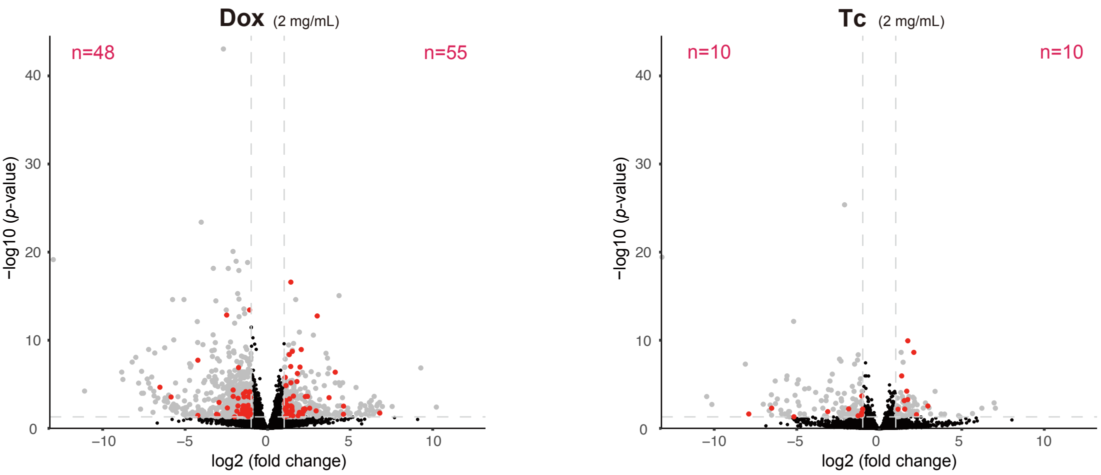

D

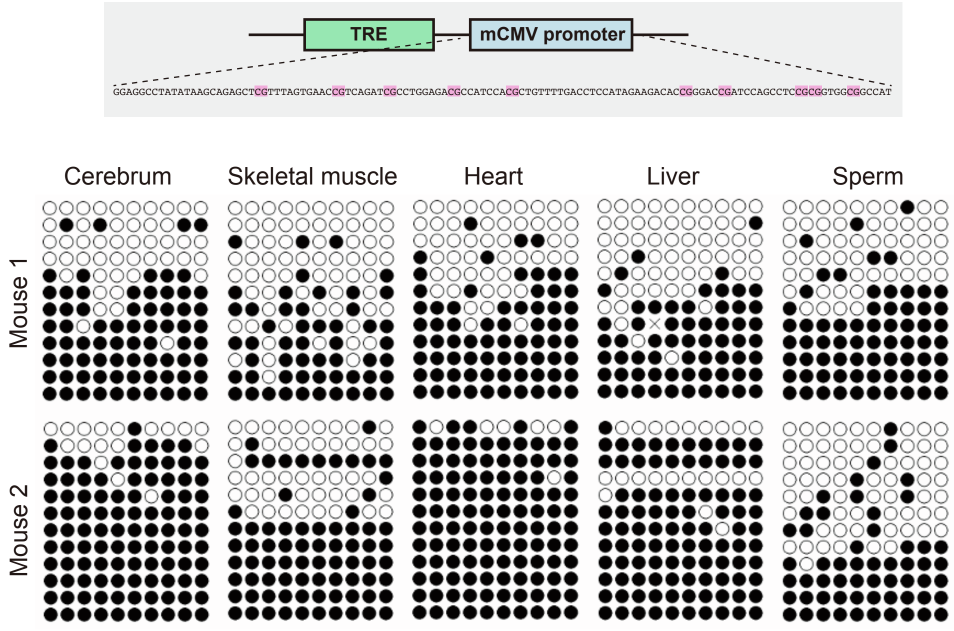

Supplemental Figure Legends

Figure S1, related to Figure 1: Cre/*loxP* recombination in PB *CAG-CreERT2* and Tet-ON/OFF mice

(A) Schematic illustration of reporter alleles to visualize Cre/*loxP* recombination.

(B) A protocol for tamoxifen administration to PB *CAG-CreERT2* reporter mice.

(C) Representative macroscopic fluorescent images of organs of PB *CAG-CreERT2* reporter mice after tamoxifen treatment. Scale bars: 5 mm.

(D) The left panel depicts a schematic illustration of Cre/*loxP* recombination for *rtTA3* expression in Tet-ON mice. Cre/*loxP* recombination is detectable in organs of Tet-ON mice after tamoxifen treatment.

(E) qPCR analysis of *rtTA3* expression in organs of Tet-ON mice. Data are presented as means ± SD of biological triplicates. Expression levels relative to those in tamoxifen-treated Tet-ON mice not administered Dox are shown.

(F) qPCR analysis of *Venus* expression in organs of Tet-ON mice. Data are presented as means ± SD of biological triplicates. Expression levels relative to those in Dox-treated Tet-ON livers are shown. ****$p<0.0001$, one-way ANOVA and Tukey's multiple-comparison test.

(G) The left panel depicts a schematic illustration of Cre/*loxP* recombination for *tTA* expression in Tet-OFF mice. Cre/*loxP* recombination is detectable in organs of Tet-OFF mice after tamoxifen treatment.

Figure S2, related to Figures 1 and 2: Microscopic analysis of VENUS expression in Tet-ON/OFF mice

(A) Representative histological images of VENUS immunostaining. VENUS expression is detectable in mesenchymal cells in the dermis, smooth muscle cells, endothelial cells, and peripheral nerve cells exclusively in Tet-OFF mice. Scale bars: 100 μm (muscle layer), 200 μm (skin and forestomach)

(B) Representative histological images of VENUS immunostaining. VENUS expression is detected in a broader range of cell types in Tet-OFF mice. Scale bars: 200 μm.

Figure S3, related to Figures 1-3: Microscopic analysis of VENUS expression in Tet-ON mice

(A) A protocol for *in vivo* induction of *Venus* expression in Tet-ON mice.

(B) Representative histological images of VENUS immunostaining. VENUS expression is mainly

observed in epithelial cells after Dox treatment and is not detected in mice without Dox administration. Scale bars: 100 μm.

Figure S4, related to Figures 1-3: Microscopic analysis of VENUS expression in Tet-OFF mice

(A) A protocol for *in vivo* induction of *Venus* expression in Tet-OFF mice.

(B) Representative histological images of VENUS immunostaining. VENUS expression is observed in a wide variety of cell types and is not detected in mice treated with Dox. Scale bars: 100 μm.

Figure S5, related to Figures 2 and 3: VENUS expression in Tet-ON/OFF adult mice and embryos

(A) Representative fluorescent images of VENUS immunofluorescence staining. The macroscopic images were obtained with the same exposure time. Scale bars: 100 μm.

(B) Fluorescence intensities of VENUS immunofluorescence staining in organs of Tet-ON/OFF mice. Fluorescence intensities were quantified using NIH ImageJ software.

(C) Schematic illustration of the *Venus* induction system in Tet-ON/OFF concepti.

(D) A protocol for *in vivo* induction of *Venus* expression in Tet-ON/OFF concepti.

(E) Representative fluorescent images of VENUS expression in Tet-ON/OFF concepti at various developmental stages. Scale bars: 25 μm (morula and blastocyst), 5 mm (E14.5 embryo).

(F) Representative histological images of VENUS immunostaining in Tet-ON E14.5 embryos. Scale bars: 200 μm.

(G) Representative VENUS immunostaining images of placental tissues at E14.5. Scale bars: 200 μm (immunostaining), 100 μm (immunofluorescence staining).

Figure S6, related to Figure 6 and Discussion: Transcriptional perturbation caused by Dox and Tc administration and DNA methylation status at the mCMV promoter in various organs of Tet-ON mice

(A) Principal component analysis of the gene expression profile of the liver. Four-week-old male mice were treated with Dox or Tc in drinking water (2 mg/mL) for 7 days. The data represent the mean values of 3 independent samples.

(B) Gene ontology terms associated with each cluster in Figure 6B.

(C) Volcano plots of RNA-seq data showing the transcriptional response to Dox or Tc administration. The data represent the mean values of 3 independent samples. Mitochondrial component genes (GO: 0005739) among differentially expressed genes (fold change>2, FDR<0.05) are labeled in red.

(D) Bisulfite sequencing analyses were conducted to determine DNA methylation status at the individual CpG sites of the mCMV promoter. White and black circles indicate non-methylated and methylated cytosine at CpG sites, respectively.

Supplemental Experimental Procedures

Vectors

piggyBac* (PB) *CAG-CreERT2

To prepare the insert, *CreERT2* with 15 bp extensions complementary to the backbone ends (2043 bp) was cloned using KOD-FX-Neo (TOYOBO). The backbone (7542 bp) was obtained using KOD-FX-Neo with a PB transposon vector carrying *CAG-EGFP-ires-NeoR* as a template. The insert and backbone of the PB vector were combined using an In-Fusion HD Cloning Kit (TaKaRa).

***Rosa26-LSL-rtTA3/tTA* targeting vector**

rtTA3 or *tTA* with the Kozak sequence (753 bp) was cloned using KOD-FX-Neo. These fragments were inserted into the pCR8-GW-TOPO vector (Thermo Fisher Scientific) and transferred into the p*Rosa26*-DEST vector (Addgene #21189) using Gateway LR Clonase II enzyme mix (Thermo Fisher Scientific), which was used as a targeting vector.

***Rosa26-tetO-Venus-ires-mCherry* targeting vector**

Venus with the Kozak sequence (726 bp) was cloned using KOD-FX-Neo. This fragment was inserted into the pCR8-GW-TOPO vector and transferred into the p*Rosa26-tetO-attR1-ccdB-attR2-ires-mCherry* vector using Gateway LR Clonase II enzyme mix, which was used as a targeting vector.

Cell culture

Embryonic stem cells (ESCs) were cultured in ESC medium, which was composed of Knockout DMEM (Gibco), 100× GlutaMAX-I (Gibco), 100× MEM non-essential amino acids (Gibco), 100 U/mL penicillin (Wako), 100 μg/mL streptomycin (Wako), 15% fetal bovine serum (Gibco), 0.1 mM mercaptoethanol (Nacalai Tesque), 1000 U/mL human leukemia inhibitory factor (Wako), 0.2 μM PD0325901 (Stemgent), and 3 μM CHIR99021 (Stemgent), on MEFs irradiated with X-rays. MEFs were cultured in MEF medium, which was composed of DMEM (Nacalai Tesque) containing 100× GlutaMAX-I, 100× MEM non-essential amino acids, 100 U/mL penicillin, 100 μg/mL streptomycin, 10% fetal bovine serum, and 0.1 mM mercaptoethanol.

Establishment of MEFs

Embryos were harvested at E14.5 and minced with razors. MEFs were cultured in MEF medium. To

induce transgene expression in MEFs, MEFs (passage 3–4) were seeded at a density of 1.5×10^5 cells/6-cm dish. The next day, Dox or Tc was added at the indicated concentration (Day 0). All cells were passaged into a 10-cm dish on Day 4 and analyses were conducted on Day 7.

Generation of chimeric mice

Eight-week-old ICR female mice (Japan SLC) were intraperitoneally injected with 7.5 U of serotropin (ASKA Animal Health). Forty-eight hours later, mice were injected with 7.5 U of gonadotropin (ASKA Pharmaceutical) and then mated with ICR male mice (Japan SLC). Two-cell fertilized eggs were collected and maintained in CARD-KSOM medium (Kyudo) to obtain blastocysts. After injection of 6–10 ESCs, blastocysts (22–26 blastocysts/mouse) were transplanted into the uteri of pseudopregnant ICR female mice (Japan SLC).

Quantification for immunofluorescence analysis

To quantify signal intensities in VENUS-positive cells, immunostained sections were randomly photographed. Three images of each tissue were processed with ImageJ software (NIH) to evaluate the region of interest. The threshold value was adjusted to 35–255 after creating green images using Split Channels. Cells within the Size 300-Infinity range were used for measurement using Analyze Particles. The brightness of each VENUS-positive cell in the image was calculated and shown as signal intensity in a dot plot.

RNA preparation

Freshly collected tissues were frozen in liquid nitrogen and ground into powder using a mortar. Total RNA was isolated from livers, hearts, and skeletal muscles using an RNeasy Fibrous Tissue Mini Kit (QIAGEN) and from cerebrums using an RNeasy Lipid Tissue Mini Kit (QIAGEN). RNA was quantified on a NanoDrop 2000 instrument.

cDNA synthesis and qPCR analysis

Five hundred nanograms of RNA was reverse-transcribed into cDNA using a PrimeScript RT Reagent Kit (TaKaRa). Quantitative real-time PCR analysis was performed using GoTaq qPCR Master Mix and CXR Reference Dye (Promega) on a StepOnePlus Real-Time PCR system (Applied Biosystems). The primers used are shown in Table S3. Transcript levels were normalized against that of *Actb*. Experiments were performed in biological triplicates.

Library preparation for RNA-seq

High-quality total RNA was isolated using NucleoSpin RNA Plus (Takara Bio). RNA-seq libraries were generated using a NEBNext Ultra II Directional RNA Library Prep Kit for Illumina (NEB), and the number of PCR cycles was minimized to avoid skewing the representation of the libraries. RNA-seq libraries were subjected to single-end sequencing (86 bp) with NextSeq500 (Illumina).

RNA-seq data analyses

Sequenced reads were trimmed to remove low-quality bases and adaptor sequences using cutadapt-4.6 (Martin, 2011). Trimmed reads were mapped to the mouse reference genome (mm10) using STAR v2.7.11ac with GENCODE vM23 (mouse) (Frankish et al., 2019). Uniquely mapped reads were summarized at the gene level using HTSeq-count v2.0.5 (Anders et al., 2015), and the expression level of each gene and differentially expressed genes were determined using DESeq2 v1.42.0 (Love et al., 2014). The Heatmap was generated based on the z-scaled mean values of normalized counts from the three biological replicates using the ComplexHeatmap v2.14.0. Clustering in the heatmap was performed using partitioning around medoids (PAM) algorithm across the genes whose expression was altered in the Dox or Tc treated mice compared to the control. A Gene Ontology (GO) enrichment analysis was performed for each cluster in the heatmap using the enrichGO function in the clusterProfiler package v4.6.2.

Bisulfite sequencing

Four-hundred nanograms of DNA was bisulfite-treated using EZ DNA Methylation-Gold Kit (ZYMO RESEARCH). PCR was performed with GO Taq Green Master Mix (Promega) with primers listed in Table S3. PCR products were cloned into the pCR4-TOPO vector (Invitrogen). After transformation into DH5α, colony PCR was performed using GO Taq Green Master Mix. After cleanup of PCR products by Exonuclease I (New England Biolabs) and Alkaline Phosphatase (Shrimp) (TaKaRa), PCR products were sequenced with M13 reverse primer. Sequencing results were evaluated using the QUMA software (RIKEN).

References

Anders, S., Pyl, P.T., and Huber, W. (2015). HTSeq--a Python framework to work with high-throughput sequencing data. Bioinformatics *31*, 166-169. 10.1093/bioinformatics/btu638.

Frankish, A., Diekhans, M., Ferreira, A.M., Johnson, R., Jungreis, I., Loveland, J., Mudge, J.M.,

Sisu, C., Wright, J., Armstrong, J., et al. (2019). GENCODE reference annotation for the human and mouse genomes. Nucleic Acids Res. *47*, D766-D773. 10.1093/nar/gky955.

Love, M.I., Huber, W., and Anders, S. (2014). Moderated estimation of fold change and dispersion for RNA-seq data with DESeq2. Genome Biol. *15*, 550. 10.1186/s13059-014-0550-8.

Martin, M. (2011). Cutadapt removes adapter sequences from high-throughput sequencing reads. 2011 *17*, 3. 10.14806/ej.17.1.200.

Table S1, related to Figure 4. In vivo studies using the Tet system (selected by keywords "Tet-ON or OFF" and "In vivo" on Pubmed)

System	Year	Author	Title	Journal	Dox conc.	Expression
Tet-ON	2023	Yoshioka H et al.,	Developmental impairments of craniofacial bone and cartilage in transgenic mice expressing FGF10	Bone Rep.	2mg/mL	Systemic (CMV-rtTA)
Tet-ON	2023	Gödecke N et al.,	A Ubiquitous Chromatin Opening Element and DNA Demethylation Facilitate Doxycycline-Controlled Expression during Differentiation and in Transgenic Mice	ACS Synth Biol.	2mg/mL	Systemic (Rosa26-rtTA)
Tet-ON	2020	Koopmans T et al.,	Smooth-muscle-derived WNT5A augments allergen-induced airway remodelling and Th2 type inflammation	Sci Rep.	2mg/mL	Smooth muscle cell (SM22-rtTA)
Tet-ON	2019	Das B and Senapati S.	Functional and mechanistic studies reveal MAGEA3 as a pro-survival factor in pancreatic cancer cells	J Exp Clin Cancer Res.	0.05mg/mL	Subcutaneous tumor
Tet-ON	2019	Huang L et al.,	Time-restricted release of multiple neurotrophic factors promotes axonal regeneration and functional recovery after peripheral nerve injury	FASEB J.	0.005mg/mL	Grafted Schwann cells
Tet-ON	2018	Alsaieedi A et al.,	Safety and efficacy of Tet-regulated IL-12 expression in cancer-specific T cells	Oncoimmunology	2mg/mL	Injected T cell
Tet-ON	2018	Chen K et al.,	Methylation-associated silencing of *miR-193a-3p* promotes ovarian cancer aggressiveness by targeting GRB7 and MAPK/ERK pathways	Theranostics	2mg/mL	Subcutaneous tumor
Tet-ON	2017	Wang Y et al.,	Reprogramming Factors Remodel Melanoma Cell Phenotype by Changing Stat3 Expression	Int J Med Sci.	5mg/mL	Subcutaneous tumor
Tet-ON	2017	Hubner EK et al.,	An *in vivo* transfection system for inducible gene expression and gene silencing in murine hepatocytes	J Gene Med.	1mg/mL	Transfected hepatocytes
Tet-ON	2016	He X et al.,	*In Vivo* magnetic resonance imaging of xenografted tumors using FTH1 reporter gene expression controlled by a tet-on switch	Oncotarget.	1 or 2mg/mL	Subcutaneous tumor
Tet-OFF	2021	Jouvet N et al.,	The Tetracycline-Controlled Transactivator (Tet-On/Off) System in β-Cells Reduces Insulin Expression and Secretion in Mice	Diabetes	1mg/mL	β cell (MIP-tTA)
Tet-OFF	2019	Hoesl C et al.,	The transmembrane protein LRIG2 increases tumor progression in skin carcinogenesis	Mol Oncol.	3mg/mL	Skin (Krt5-tTA)
Tet-OFF	2019	Guo Y et al.,	Inducible cardiac-specific overexpression of cyclooxygenase-2 (COX-2) confers resistance to ischemia/reperfusion injury	Basic Res Cardiol.	2mg/mL	Cardiomyocyte (α-MyHC-tTA)
Tet-OFF	2018	Wondimu EB et	Elf3 Contributes to Cartilage Degradation in vivo in a Surgical Model of Post-	Sci Rep.	1mg/mL	Cartilage (Comp-

		al.,	Traumatic Osteoarthritis			tTA)
Tet-OFF	2018	Kong B et al.,	Fibroblast Growth Factor 15–Dependent and Bile Acid–Independent Promotion of Liver Regeneration in Mice	Hepatology	2mg/mL	Liver, Intestine (Fabp-tTA)
Tet-OFF	2018	Joshi SS et al.,	Characterization of a new, inducible transgenic mouse model with GFP expression in melanocytes and their precursors	Gene Expr Patterns.	2mg/mL	Melanocyte stem cell (Dct-tTA)
Tet-OFF	2016	Santacatterina F et al.,	Down-regulation of oxidative phosphorylation in the liver by expression of the ATPase inhibitory factor 1 induces a tumor-promoter metabolic state	Oncotarget	2mg/mL	Liver (Lap-tTA)
Tet-OFF	2015	Shuen WH et al.,	Novel lentiviral-inducible transgene expression systems and versatile single-plasmid reporters for in vitro and in vivo cancer biology studies	Cancer Gene Ther.	0.2mg/mL	Subcutaneous tumor
Tet-OFF	2012	Orlando UD et al.,	The functional interaction between Acyl-CoA synthetase 4, 5-lipooxygenase and cyclooxygenase-2 controls tumor growth: a novel therapeutic target	PLoS One	2mg/mL	Subcutaneous tumor
Tet-OFF	2012	Yoshida M et al.,	Nuclear translocation of pro-amphiregulin induces chemoresistance in gastric cancer	Cancer Sci.	1mg/mL	Subcutaneous tumor

Table S2, related to Figure 4. In vivo reprogramming studies using the Tet system

System	Year	Author	Title	Journal	Dox conc.	Expression
Tet-ON	2013	Abad M et al.,	Reprogramming *in vivo* produces teratomas and iPS cells with totipotency features	Nature	0.2 or 1mg/mL	Systemic
Tet-ON	2014	Ohnishi K et al.,	Premature Termination of Reprogramming In Vivo Leads to Cancer Development through Altered Epigenetic Regulation	Cell	2mg/mL	Systemic
Tet-ON	2016	Mosterio L et al.,	Tissue damage and senescence provide critical signals for cellular reprogramming in vivo	Science	0.2mg/mL	Systemic
Tet-ON	2016	Ocampo A et al.,	In Vivo Amelioration of Age-Associated Hallmarks by Partial Reprogramming	Cell	1mg/mL	Systemic
Tet-ON	2018	Shibata H et al.,	In vivo reprogramming drives *Kras*-induced cancer development	Nat Commun.	2mg/mL	Pancreas (Pdx1-ires-Cre)
Tet-ON	2018	Doeser M et al.,	Reduction of Fibrosis and Scar Formation by Partial Reprogramming In Vivo	Stem Cells	2mg/mL	Skin (Gauze pad)
Tet-ON	2020	Rodríguez-Matellán A et al.,	In Vivo Reprogramming Ameliorates Aging Features in Dentate Gyrus Cells and Improves Memory in Mice	Stem Cell Reports	2mg/mL	Systemic
Tet-ON	2020	Lu Y et al.,	Reprogramming to recover youthful epigenetic information and restore vision	Nature	2mg/mL	Retina (AAV2-rtTA)
Tet-ON	2021	Wang C et al.,	In vivo partial reprogramming of myofibers promotes muscle regeneration by remodeling the stem cell niche	Nat Commun.	1mg/mL	Myofiber (Acta1-Cre)
Tet-ON	2021	Chen Y et al.,	Reversible reprogramming of cardiomyocytes to a fetal state drives heart regeneration in mice	Science	5mg/mL	Cardiomyocyte (Xmlc2-Cre)
Tet-ON	2021	Taguchi J et al.,	DMRT1-mediated reprogramming drives development of cancer resembling human germ cell tumors with features of totipotency	Nat Commun.	2mg/mL	Kidney (Pax8-Cre), Pancreas (Pdx1-Cre)
Tet-ON	2022	Chondronasious D et al.,	Deciphering the roadmap of *in vivo* reprogramming toward pluripotency	Stem Cell Reports	1mg/mL	Systemic
Tet-ON	2022	Browder KC et al.,	In vivo partial reprogramming alters age-associated molecular changes during physiological aging in mice	Nat Aging	1mg/mL	Systemic
Tet-ON	2022	Hishida T et al.,	*In vivo* partial cellular reprogramming enhances liver plasticity and regeneration	Cell Rep.	0.1mg/mL	Liver (Alb-Cre)

Tet-ON	2022	Alle Q et al.,	A single short reprogramming early in life initiates and propagates an epigenetically related mechanism improving fitness and promoting an increased healthy lifespan	Aging Cell	0.2-0.5mg/mL	Systemic
Tet-ON	2023	Kim J et al.,	Partial in vivo reprogramming enables injury-free intestinal regeneration via autonomous *Ptgs1* induction	Sci Adv.	0.15mg/mL	Systemic
Tet-ON	2023	Parras A et al.,	In vivo reprogramming leads to premature death linked to hepatic and intestinal failure	Nat Aging	1mg/mL	Systemic, Liver (Alb-Cre), Small intestine (Vil1-Cre)
Tet-ON	2024	Xu L et al.,	Restoration of neuronal progenitors by partial reprogramming in the aged neurogenic niche	Nat Aging	1mg/mL	Systemic
Tet-OFF	2020	Lu Y et al.,	Reprogramming to recover youthful epigenetic information and restore vision	Nature	2mg/mL	Retina (AAV2-tTA)
Tet-OFF	2024	Antón-Fernández A et al.,	In vivo cyclic overexpression of Yamanaka factors restricted to neurons reverses age-associated phenotypes and enhances memory performance	Commun Biol.	2mg/mL	Neuron (α-CaMKII-tTA)

Table S3, related to Figures 3-5, S1, and S6. Primers used in this study

Primer for qPCR	Sequence 5'→3'
Actb FW	GCCAACCGTGAAAAGATGAC
Actb RV	TCCGGAGTCCATCACAATG
rtTA FW	ATGTCTAGACTGGACAAGAGCAAAGT
rtTA RV	CTTGTTCTTCACGTGCCAGTACAGG
Venus FW	CTACCCCGACCACATGAAGCAG
Venus RV	GTCGCCCTCGAACTTCACCTC

Primer for bisulfite-seq	Sequence 5'→3'
mCMV bisulfite-seq FW	GTGGGAGGTTTATATAAGTAGAGTT
mCMV bisulfite-seq RV	AAACCTACTTTTTTATACAAACTTATAATAAC
